# Tin Oxide Based Hybrid Nanostructures for Efficient Gas Sensing

**DOI:** 10.3390/molecules27207038

**Published:** 2022-10-18

**Authors:** Nayeem Ahmad Pandit, Tokeer Ahmad

**Affiliations:** Nanochemistry Laboratory, Department of Chemistry Jamia Millia Islamia, New Delhi 110025, India

**Keywords:** gas sensor, gas sensing, tin oxide, nanostructures, composite, hybrid

## Abstract

Tin oxide as a semiconductor metal oxide has revealed great potential in the field of gas sensing due to its porous structure and reduced size. Especially for tin oxide and its composites, inherent properties such as high surface areas and their unique semiconducting properties with tunable band gaps make them compelling for sensing applications. In combination with the general benefits of metal oxide nanomaterials, the incorporation of metal oxides into metal oxide nanoparticles is a new approach that has dramatically improved the sensing performance of these materials due to the synergistic effects. This review aims to comprehend the sensing mechanisms and the synergistic effects of tin oxide and its composites in achieving high selectivity, high sensitivity and rapid response speed which will be addressed with a full summary. The review further vehemently highlights the advances in tin oxide and its composites in the gas sensing field. Further, the structural components, structural features and surface chemistry involved in the gas sensing are also explained. In addition, this review discusses the SnO_2_ metal oxide and its composites and unravels the complications in achieving high selectivity, high sensitivity and rapid response speed. The review begins with the gas sensing mechanisms, which are followed by the synthesis methods. Further key results and discussions of previous studies on tin metal oxide and its composites are also discussed. Moreover, achievements in recent research on tin oxide and its composites for sensor applications are then comprehensively compiled. Finally, the challenges and scope for future developments are discussed.

## 1. Introduction

Metal oxide nanoparticles represent a field of materials chemistry and have attracted considerable attention due to their potential applications in domestic, industrial and commercial fields as sensors due to their many significant features such as easy production, low cost and compact size [1,2]. The potential implications of metal oxides in fields such as energy storage, catalysis, medicine, informational technology and gas sensing have driven much research attention to the development of synthetic pathways towards their nanostructure fabrication. Due to the reduced size and increased surface-to-volume ratio, the nano-sized compounds have shown applications in different fields such as gas sensing [3,4,5,6], catalysis [7,8,9,10,11], lithium-ion batteries [12,13,14] and dye-sensitized solar cells [15,16,17,18]. Among various applications of nanomaterials, gas sensing has attracted much attention from the scientific community due to the increased demand for efficient sensors for defense, environmental applications [19], exhaust gas determination in automobiles [20], leakage determination in chemical plants [21], product quality assurance in food companies [22], etc. One of the most prominent applications of gas sensors is the detection of harmful gases present in our environment and their precise monitoring beyond a certain limit, which has become the most challenging aspect for humankind in this ever-polluted environment. Thus, the preparation of gas sensors is ever-increasingly demanded, particularly for those sensors which can sense dreadful gases even at very small ppm levels that are very toxic not only to humans but also to other organisms. Among the various classes of nanomaterials such as metal sulfides, metal phosphides and metal oxides, the metal oxides as gas sensors are efficient as compared to their counterparts. The metal oxides have fast response, excellent sensitivity and recovery and above all low cost, while the sulfides and phosphides are lacking in these properties. Several advanced nanomaterials are known for nanocatalysis [23,24,25,26], and rich fabrication techniques are known [27,28,29] for their stabilization. The focus in designing the metal oxide nanostructures and their composites is on enhancing their properties of gas sensing such as response speed, selectivity and sensitivity [30].

The performance of tin oxide nanoparticles as sensors is greatly influenced by structure and morphology which otherwise create a great hindrance to achieving highly sensitive properties of gas sensors based on bulk materials. The close intimate link between the chosen synthetic pathway and morphology could allow the preparation of energy-efficient metal oxide nanoparticles whose properties can be exploited in technologically important areas of sensors [31,32,33,34,35]. N-type metal oxides are the most dynamic metal oxides used as gas sensors and are being thoroughly investigated. Among them, SnO_2_ as an n-type semiconductor has received many considerations in environmental monitoring, catalysis, transparent conducting films, lithium rechargeable batteries, biochemical sensors, ultra-sensitive gas sensors and dye-sensitized solar cells [36,37,38,39,40,41,42]. In addition, SnO_2_ as an alcohol sensor has useful applications in fields such as the food industry and breath analysis that are very beneficial for human health [43]. Nowadays, much work is being performed for synthesizing different types of nanostructures with a wide range of morphologies such as hollow spheres, core–shell, microspheres, nanospheres, nanotubes and nanowires for the sensing of ethanol, H_2_ and methanol [44,45,46,47,48]. Among these morphologically different nanostructures, ethanol shows better sensing results as compared to H_2_ and methanol. For example, Vuong et al. synthesized SnO_2_ microspheres, nanorods and nanoflowers via a simple hydrothermal route [49]. Likewise, Zhang et al. studied mesoporous tin dioxide nanopowder-based sensors to selectively detect ethanol vapor [50]. Similarly, Sahay et al. prepared monocrystalline SnO_2_ nanostructures and studied AC transport properties [51].

An important parameter and a distinguishing feature of an efficient metal oxide gas sensor is its sensing property, which includes sensitivity, selectivity and response speed, whose details are largely unexplored and represent our focus in this review. The sensing property is remarkably known for a large number of gas detecting systems in various types of control processes and laboratory analysis [52,53,54]. Additionally, even if a number of reviews have already been written on metal oxides as gas sensors [55,56,57], much still needs to be done with respect to their particle size and morphology in order to enhance their sensing properties, which we will address in this comprehensive review. Further, it has been concluded that a large surface-to-volume ratio of SnO_2_ and its different composites will ensure enhanced surface area available for the target gas interactions, as a result of which the realization of high sensitivity even at low temperatures can be obtained. As a consequence of this, the materials with high specific surface area and specific surface morphology will manifest the presence of sufficient active sites that can selectively and effectively interact with the target gas, which can consequently improve the sensing performance of a material, including its sensitivity and selectivity [58]. Although many reviews have already been written on the metal oxides as gas sensors, no systematic and comprehensive effort has been made towards their lack of stability and limited selectivity which has resulted in the gas response’s “long-term spoiling”. Hence, in this review, our main focus will be on these two parameters. Even though various types of laboratory analysis and control processes have been employed for gas detecting systems [52,53,54], high-selectivity, rapid-response speed and high-sensitivity gas sensors are still the need of the hour to improve the levels of gas sensing even at very minute levels. Hence, in this review, we will provide a holistic overview of metal oxide nanostructures and composites for gas sensing applications with precise monitoring, keenly observing the changes incorporated by doping, particle size and morphology [59,60,61,62]. Herein, we comprehensively review the literature of a particular metal oxide, i.e., SnO_2_, and its composites to understand the fundamental gas sensing mechanisms and current progress in the field of gas detection. In addition, we will discuss the necessary structural components, sensing mechanisms and the development of composite architectures for the better development of sensing pathways which will have a better and insightful enhancement in their sensing response. Hence in this review, we will implement a holistic and integrated approach and consider the inroads made in the earlier approaches to metal oxide nanoparticles and the forays to develop and obtain a deep insight into the metal oxide gas sensors with challenges and future directions to unravel the complications in achieving high selectivity, high sensitivity and rapid response speed of metal oxides by various synthetic procedures [62].

## 2. Gas Sensing Mechanism

**Figure 1:**
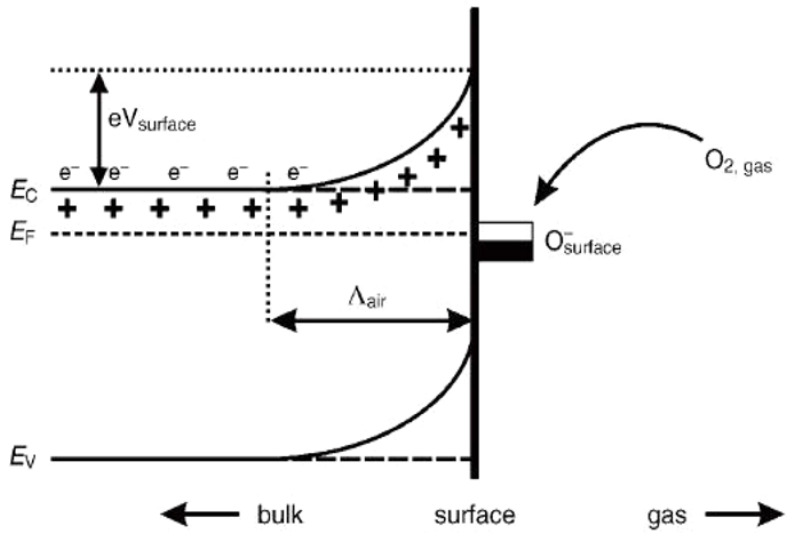
Simplified model illustrating band bending in a wide band gap semiconductor after chemisorption of charged species (here the ionosorption of oxygen) on surface sites. EC, EV and EF denote the energy of the conduction band, valence band and Fermi level, respectively, while Ʌ_air_ denotes the thickness of the space charge layer, and eV_surface_ denotes the potential barrier. The conducting electrons are represented by e^−^, and + represents the donor sites. (Reprinted with permission from [64], Copyright 2003, Elsevier.).

In order to design highly efficient metal oxide gas sensors, it is necessary to understand the underlying sensing mechanism responsible for the sensing property of the materials. Besides understanding the mechanism, it is also important to have an in-depth knowledge of the factors which influence these sensing properties of the materials. Although the causes of gas response and its exact fundamental mechanism are still controversial, essentially the trapping of electrons at adsorbed molecules and the band bending induced due to the effect of charged molecules are the prime factors responsible for a change in conductivity. Here, the sensing mechanism of metal oxides with n-type in air is given with reference to SnO_2_. Usually, the adsorption of oxygen in air takes place on the surface of the SnO_2_ sensing film. The oxygen species adsorbed on the surface of SnO_2_ can pull electrons from the inner surface of the SnO_2_ film. At an operating temperature of 300–450 °C [63], the O^−^ is believed to be dominant, and this range is considered as its working temperature. As shown in Figure 1 [64], the conduction band (Ec) electrons are extracted by the adsorption of O_2_ molecules which are present on the metal oxide surface, and these electrons in the form of ions are then trapped at the surface, which leads to the formation of a region which is devoid of electrons, generally called an electron-depleted region, and a band bending in which the electron-deficient region is the so-called space charge layer whose length of thickness is the region of band bending. Consequently, there is a decrease in the reaction between reducing gases and oxygen species that results in the reversal of the band bending, which increases the conductivity of gas sensors for most metal oxides. Figure 1 depicts the mechanism of conduction when depletion regions are smaller than the grain size, suitable only for n-type semiconducting metal oxides.

**Figure 2:**
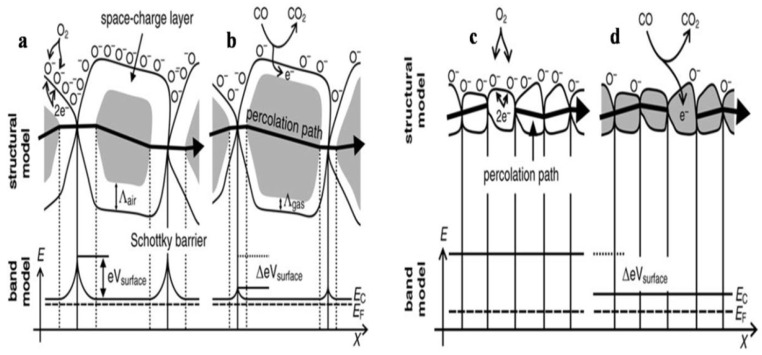
Structural and band model showing the role of intergranular contact regions in determining the conductance over a polycrystalline metal oxide semiconductor: (**a**) initial state and (**b**) effect of CO on Λ_air_ and eV_surface_ for large grains; structural and band model for particles with D <2 Λ_air_ leading to the so-called “flat-band” case: (**c**) the initial state and (**d**) the effect of CO on the position of the conduction band E_c_ (Reprinted with permission from [64] Copyright 2003, Elsevier).

The stoichiometry of the overall metal oxide surface plays a decisive role in the surface conductivity phenomena of gas sensors. The increase in surface conductivity is a result of donors which are oxygen vacancies, and when the electrons adsorb oxygen ions, the surface conductivity diminishes and acts as a surface acceptor. Further when reference gas is exposed with or without CO, the CO is oxidized by O^−^ upon exposure of gas sensors to the reference gas with CO and thus results in the release of electrons to the bulk medium. Moreover, the space charge layer decreases in thickness as a result of a fall in the surface O^–^ number, and because of that, the Schottky barrier between the two grains is lowered, and ultimately the electrons can conduct in sensing layers through different grains. The conductive mechanism based on the structural and band model is illustrated by Figure 2a–d.

## 3. Synthesis of Tin Oxide and Its Composites

With the advent of nanochemistry, research in the fields of synthesis and characterization of nanoparticles and nanostructures has received considerable attention, and the high-technological applicability of nanoparticles and nanostructures makes them potentially relevant in different fields. Nowadays, nanomaterials are widely used not only for biological and environmental applications but also for sensing and electronic applications. Among the various classes of nanomaterials such as metal sulfides, metal phosphides and metal oxides, the metal oxide nanostructures possess some superior and peculiar characteristics due to their unique chemical and physical properties as a result of which highly efficient nanodevices are fabricated from them and they are surprisingly valuable in gas sensing applications. The metal oxide nanomaterials, being superior, find their use in different fields such as catalysis, medicine, sensing and electronic devices. Due to their large number of applications, researchers have developed various synthetic strategies to produce metal oxide nanostructures with tailored geometries [65,66,67,68] and utilize them for a wide variety of applications. This section of the review will have a simplistic and systematic overview of the different synthetic strategies involved and the effect of these approaches on the reaction conditions and the final product obtained. Synthesis of different nanomaterials such as metal oxides, metal phosphides and metal sulfides, particularly the metal oxides with desired size, shape and crystal morphology, has been the cause of concern ever since the inception of different synthetic methods and has been regarded as very crucial in different fields according to applicability; therefore, many routes or methods have been developed by different researchers in order to obtain the best metal oxides with enhanced sensing properties. It has been observed that physical and chemical conditions and the method of synthesis play a decisive role in determining the shape, crystal structure, size, morphology and other properties of the final product. Thus, on the basis of the present literature survey, various types of chemical methods such as hydrothermal, solvothermal, polymeric citrate precursor, co-precipitation, microemulsion and sol–gel methods and other methods such as ion exchange, photo deposition, electrochemical and other solid state techniques have been employed to synthesize different types of metal oxides for varied purposes. Some of the commonly employed routes for the synthesis of metal oxides are discussed in this review.

## 4. Hydrothermal Method

**Figure 3:**
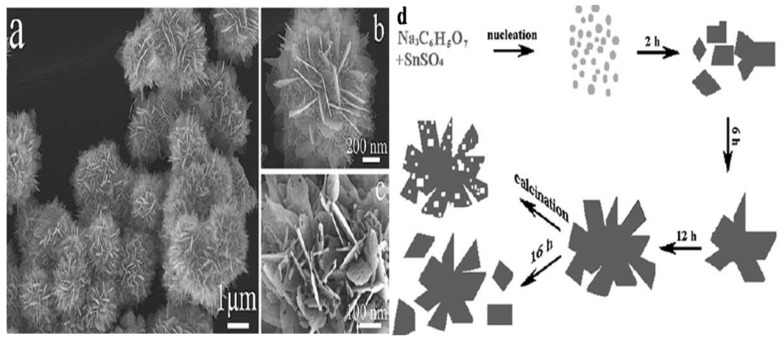
(**a**) SEM image of SnO_2_ porous 3D architectures after annealing the precipitates at 600 °C for 2 h; (**b**,**c**) magnified SEM images; (**d**) schematic representation of the process of formation of SnO_2_ hierarchical nanosheets. (Reprinted with permission from [69]. Copyright 2011, the Royal Society of Chemistry, London).

The hydrothermal method is one of the most rapidly and commonly growing methods for the synthesis of metal oxide nanoparticles, especially tin oxide nanoparticles. This method of synthesis has received more attention and increased in significance in the last few years because of certain added advantages such as the ease of controlling reaction conditions (e.g., pressure, temperature, pH), and metal oxides of varied morphologies are produced by altering the reaction conditions. Sun et al. [69] used a facile one-step hydrothermal route to synthesize SnO_2_ nanostructures by calcining precipitates composed of 2D nanosheets with high porosity. The morphological evolution of SnO_2_ samples with time and the entire possible growth mechanism were ascribed to the self-assembly and nucleation of building blocks. The final morphology, i.e., random porous structures in the nanosheets of the product, was attributed to the concentration of the precursor. The experimental results reveal a possible growth mechanism for the as-prepared SnO_2_ nanostructures. Moreover, it was observed that the SnO_2_ nanostructures which were annealed at 600 °C for 2 h showed higher gas sensing properties as compared to the sensor SnO_2_ nanoparticles prepared conventionally. This change in sensing was ascribed to the unique flower-like structures, as shown in Figure 3a–c, which usually facilitate mass transportation and gas diffusion in sensing materials. Figure 3d shows the pictorial representation process for the formation of SnO_2_ hierarchical nanosheets.

**Figure 4:**
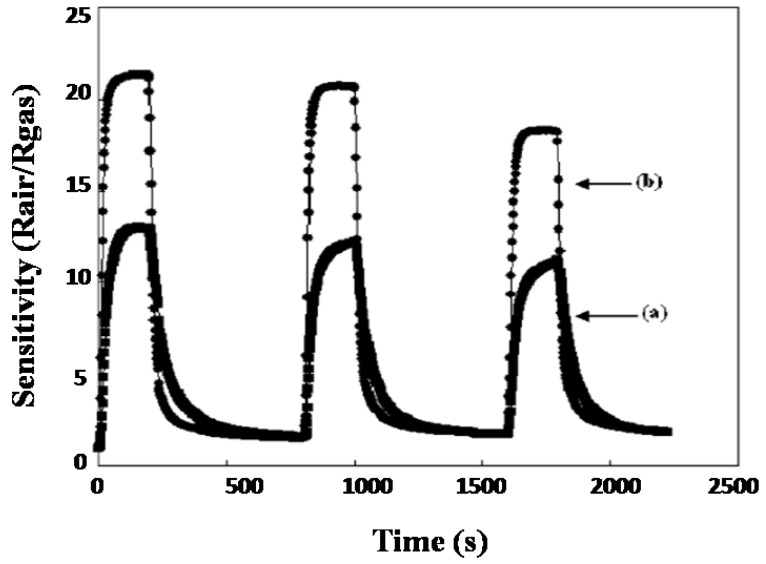
Repetitive response of the (**a**) as-synthesized and (**b**) thermally treated SnO_2_ to ethanol (25 ppm) at 220 °C. (Reprinted with permission from [70]. Copyright 2007, American Chemical Society, Washington, DC, USA).

Chiu et al. [70] developed a hydrothermal method for the synthesis of nanocrystalline SnO_2_ nanoparticles with an average grain size of 3.0 ± 0.5 nm. As-synthesized SnO_2_ particles were annealed at 300 °C for 1 h under 10% H_2_/Ar. An atomic ratio of 2.3/1 (O/Sn) was found for the SnO_2_ nanoparticles prepared thermally, and an atomic ratio of 1.2/1 was found for the as-synthesized SnO_2_ nanoparticles. For the thermally treated SnO_2_, a surface area of about 92 m^2^/g was observed as compared to the 130 m^2^/g for the SnO_2_ nanoparticles synthesized at 150 °C with rough undefined morphology. In the as-synthesized SnO_2_, the oxygen ions are replaced by the chloride ions; the thermally treated SnO_2_ acts as a better sensor for ethanol compared to the as-synthesized SnO_2_. For the as-synthesized SnO_2_ sensor, the sensitivity of SnO_2_ nanoparticles can be enhanced by heating simply for 5 min at 350 °C to remove Cl^−^ partly. Consequently, efficient gas sensing in response to ethanol was revealed for the SnO_2_ gas sensor with a detection limit of as low as 1.7 ppm. Figure 4 shows the comparative response of SnO_2_ nanoparticles to ethanol before annealing and after annealing.

**Figure 5:**
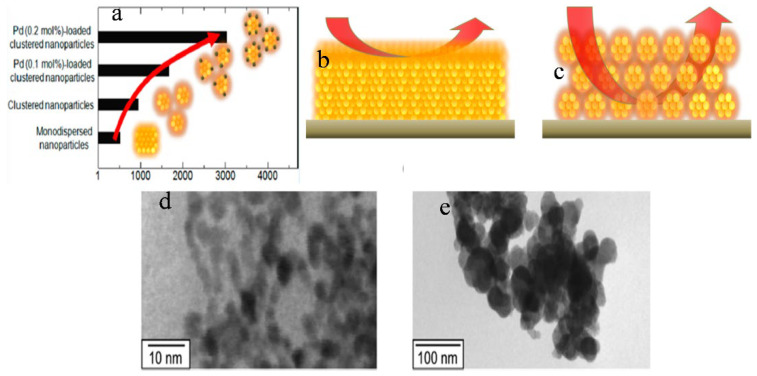
(**a**) Sensor response of different nanoparticles to toluene; (**b**,**c**) represent the gas diffusion in films made with monodispersed nanoparticles and clustered nanoparticles, respectively; (**d**) and (**e**) are the TEM images of SnO_2_ nanoparticles at pH 10.6 and pH 9.3, respectively. (Reprinted with permission from [71]. Copyright 2014, American Chemical Society, Washington, DC, USA).

**Figure 6:**
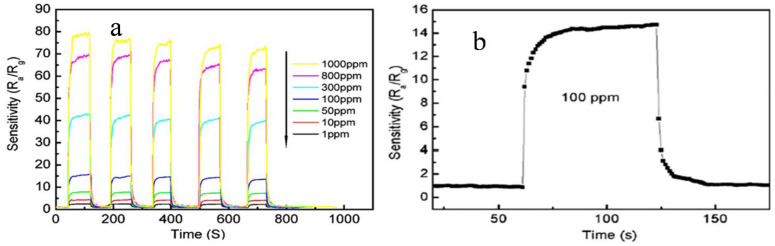
(**a**) Schematic representation of the different sensing characteristics of SnO_2_ hollow spheres upon exposure to different ethanol concentrations ranging from 1 to 1000 ppm; (**b**) sensor response and recovery times of different SnO_2_ hollow spheres to 100 ppm of ethanol for one cycle (Reprinted with permission from [38]. Copyright 2008, Elsevier).

Suematsu et al. [71] used a hydrothermal route to synthesize SnO_2_ clustered nanoparticles from SnO_2_.nH_2_O where preformed nanocrystals (ca. 5 nm) agglomerated at pH 9.3 to form large secondary nanocrystals (ca. 45 nm). Adding Pd-[(NH_3_)_2_(NO_2_)_2_] to the precursor solution resulted in the formation of Pd-loaded clusters of SnO_2_ nanoparticles. Toluene was sensed by the application of highly fabricated films of clustered SnO_2_ nanoparticles with spherical morphology. The spin coating method was used to develop highly porous gas sensing films by loading Pd with the clustered SnO_2_ particles. An improved response of sensors at 300 °C to H_2_ and CO was observed for the sensor devices using the porous films. The sensor response was increased by increasing the film porosity, which enhanced the diffusivity through the sensing films. Note that the sensor response was further increased by loading Pd onto the clustered nanospheres as a result of electrical and catalytic sensitization effects. More importantly, the Pd-loaded SnO_2_ nanoparticles showed increased toluene sensitivity due to an increase in gas diffusivity through the sensing film which helped in its detection at very low ppb levels, as shown in Figure 5a–c. From TEM results, it was concluded that the agglomeration of nanoparticles was a result of a decrease in the pH of the solution forming larger secondary nanospheres, as shown in Figure 5d,e. Tan et al. [38] demonstrated a simple hydrothermal method to synthesize SnO_2_ hollow sphere nanoparticles using carbon microspheres as templates with the decomposition of SnCl_4_. The obtained nanospheres with hollow spherical morphology have a size of 100 nm, and a significant number of SnO_2_ nanospheres have a diameter of nearly 6 nm. The obtained sensitivity of SnO_2_-based hollow nanospheres was as high as 75 to 1000 ppm for ethanol, and the response and recovery times were 4 and 10 s, respectively as shown in Figure 6. The increase in sensing property can be ascribed to the porous structure of hollow SnO_2_ nanospheres and the small size of nanospheres. It was revealed that SnO_2_ nanospheres can act as promising materials with fast response and recovery for the fabrication of gas sensors.

**Figure 7:**
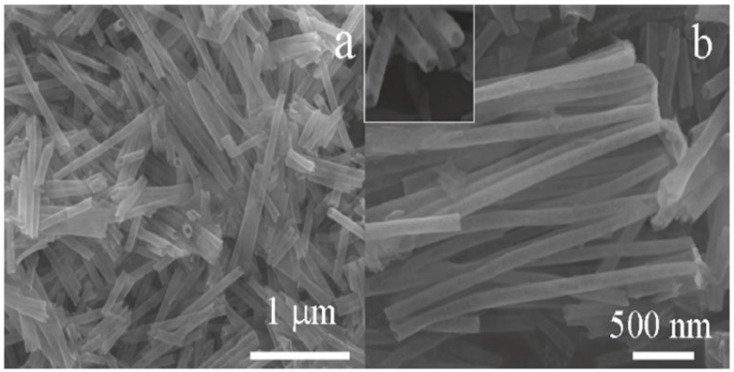
SEM images of the as-prepared SnO_2_ nanotubes: (**a**) overview and (**b**) magnified image showing the open ends of nanotubes. (Reprinted with permission from [72]. Copyright 2011, American Chemical Society, Washington, DC, USA).

Shi et al. [72] prepared SnO_2_ nanotubes on a hard template of polycarbonate by employing a hydrothermal route at low temperatures. The obtained nanostructures with nanotube morphology were found to be a few nanometers in size with fine particles, as shown in Figure 7a,b. It was observed that as the reaction temperature increased gradually, the size of the SnO_2_ nanostructures also showed an increase. The SnO_2_ nanocrystals showed that the band gap of these nanostructures increased from 3.75 eV with a particle size of 5.6 nm to 3.99 eV with a particle size of 3.3 nm. The results showed that the SnO_2_ nanotubes can have potential applications for gas sensors with enhanced gas sensitivity.

**Figure 8:**
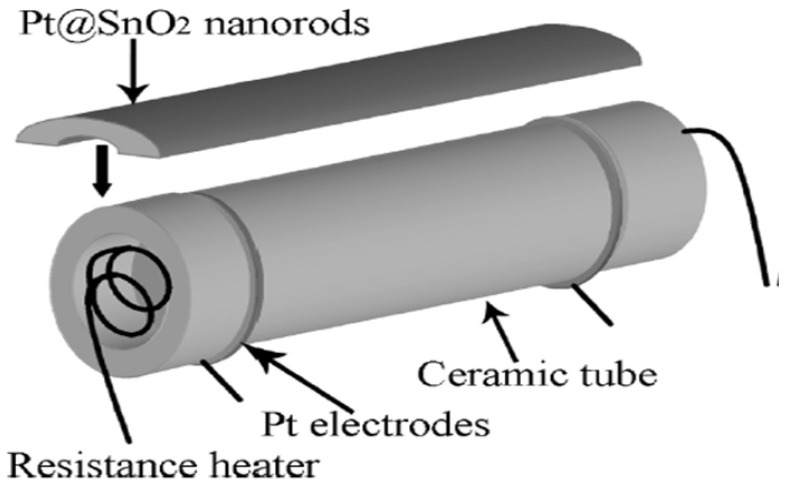
Schematic diagram showing Pt@SnO_2_ nanorod-based gas sensor. (Reprinted with permission from [73]. Copyright 2010, American Chemical Society, Washington, DC, USA).

Xue et al. [73] employed a simple one-step hydrothermal route and successfully synthesized a highly sensitive Pt@SnO_2_ nanorod-based gas sensor, as represented pictorially in Figure 8. The morphology of synthesized nanoparticles obtained were nanorods with microstructures. At a high temperature of about 300 °C, upon exposure to 200 ppm of ethanol, the sensor sensitivity reached 39.5. This can be attributed to the influence of electrical and chemical contributions of Pt, because of which the sensor displays high gas sensitivity. Further, when the sensor is heated to 200 °C, opposite variations of resistances are observed, which may be attributed to the surface oxygen ions having different temperature-dependence. The results reveal that the synthesized nanostructures are potential sensors with high performance capacity.

**Figure 9:**
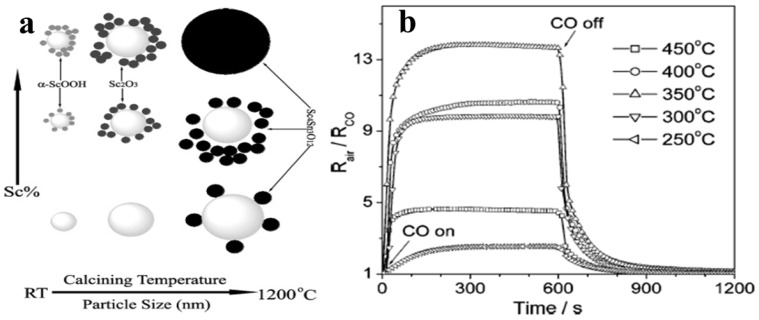
(**a**) Phasic dependence of the (SnO_2_)_1−X_ (Sc_2_O_3_)_X_ (X = 0.002–0.6) nanocrystallites upon the calcination temperature/particle size; (**b**) effect of operation temperature on the sensitivity of CO in 1000 ppm of the as-sintered (SnO_2_)_0.90_ (Sc_2_O_3_)_0.10_ pellet. (Reprinted with permission from [74]. Copyright 2005, American Chemical Society, Washington, DC, USA).

Xu et al. [74] for the first time synthesized Scandia-doped tin oxide powders in the presence of urea by a two-step hydrothermal method (produces weakly agglomerated nanocrystallites) followed by calcination between 500 and 1200 °C. When Sc_2_O_3_ is added as a dopant in appropriate amounts in nanosized SnO_2_, as was revealed by the textural studies, it can retard grain growth and stabilize the surface area to withstand high calcination temperatures below 1000 °C with discrete undefined morphology. The sensing measurements of CO gas reveal that sensitivity of SnO_2_ can improve significantly when Sc is incorporated at the surface of the nanocrystal, and at 800 °C, a pellet sample with 10 mol% of Scandia content displays enhanced sensing properties in response to CO in the operating temperature range of 300–400 °C. Figure 9a,b shows the variation of phases with Sc percentage and calcining temperature and the sensitivity of as-synthesized Sc doped SnO_2_ at different operating temperatures, respectively.

**Figure 10:**
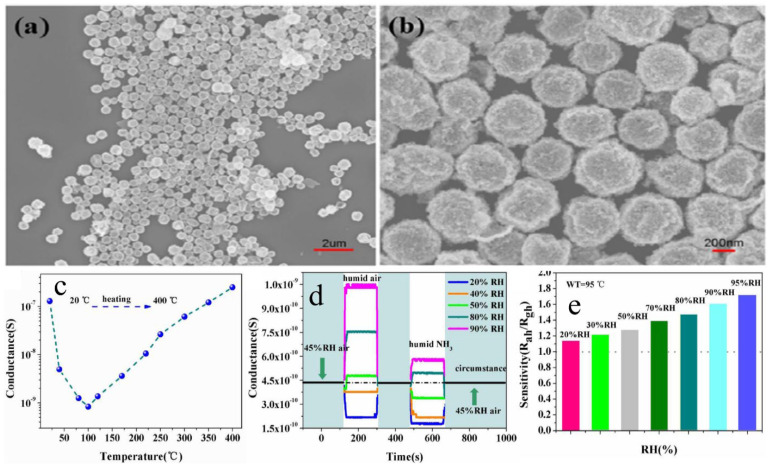
(**a**,**b**) SEM of the WO_3_−SnO_2_ hollow nanospheres. (**c**) Temperature−dependent conductance of the gas sensor. (**d**) Effect of humidity on the NH_3_ sensing at 95 °C. (**e**) Improvement in gas sensitivity to 5000 ppm ethanol by increasing surrounding humidity from 20 to 95% RH. (Reprinted with permission from [75]. Copyright 2015, American Chemical Society, Washington, DC, USA).

**Figure 11:**
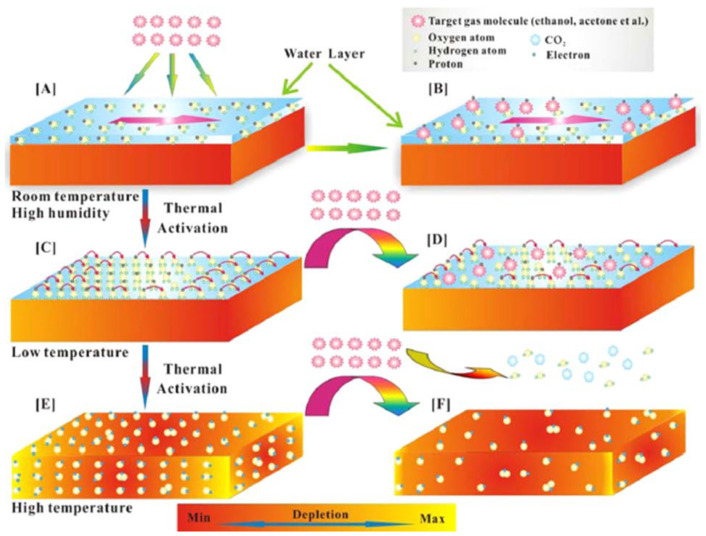
Schematic diagram showing the sensing mechanism of sensors at different working conditions: (**A**,**B**) high temperature and high humidity, (**C**,**D**) low temperature (a slight thermal shock to the sensor) and (**E**,**F**) high temperature. (Reprinted with permission from [75]. Copyright 2015, American Chemical Society, Washington, DC, USA).

Li et al. [75] synthesized WO_3_ and SnO_2_ hollow spheres by a simple hydrothermal method followed by calcination. For synthesis, 0.5 mmol of Na_2_SnO_3_, 5 g of glucose and 1 mmol of Na_2_WO_4_ were mixed in 50 mL of distilled water. After this, the solution was then transferred into a stainless Teflon-lined autoclave and stirred for several minutes, sealed and heated at a temperature of 200 °C for 20 h. In this approach, extrinsic sensing behaviors with WO_3_ and SnO_2_ hollow-sphere-based gas sensors were observed. These responses obtained were treated as pseudo-sensing responses, and these results were considered as the interactions between adsorbed water and target gas. With the increase in temperature, the response of the pseudo-p-type sensor to ethanol can be converted into normal n-type as a result of transitions of sensing mechanisms. The WO_3_-SnO_2_ composites showed an enhanced sensing property which accounts for the extrinsic sensing behaviors. The results reveal that humidity has a counterproductive effect on gas sensing, and they open up a new promising way to produce gas sensors with reduced operation temperature or synthesize gas sensors at room temperature with little power consumption. SEM results reveal that the morphology of the synthesized samples was hollow spherical, roughly uniform with good monodispersity as shown in Figure 10a,b, whereas Figure 10c–e reveal the temperature dependence, effect of humidity on ammonia sensing and improvement in gas sensitivity to 5000 ppm ethanol of the as-developed SnO_2_/WO_3_ based sensor. Similarly, Figure 11 shows the mechanism of gas sensing over the surface of SnO_2_/WO_3_ at different operating temperatures.

## 5. Polymeric Citrate Precursor Method

**Figure 12:**
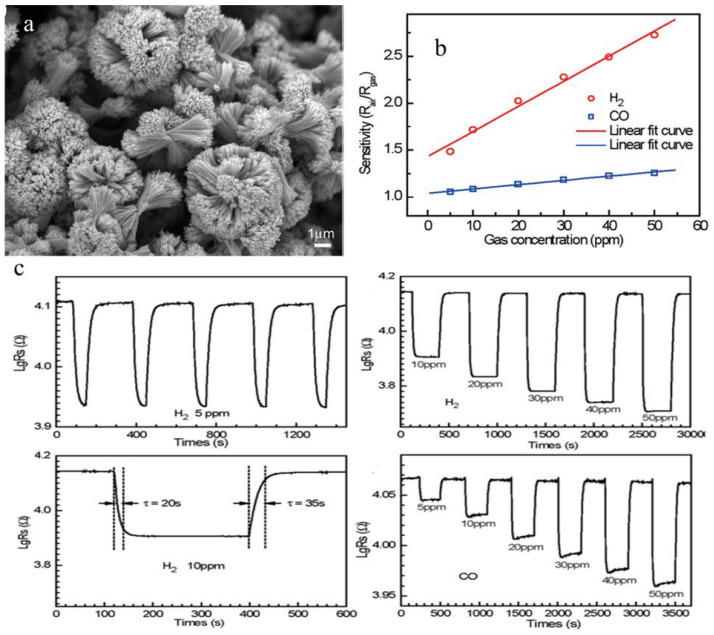
(**a**) SEM image of SnO_2_ microstructure. (**b**) Linear dependence relation between resistance response sensitivity and gas concentration. (**c**) Exposure of SnO_2_ microstructure to H_2_ for gas sensing depicts the change in concentration of H_2_ and its effect on sensor response, the response and recovery times for H_2_ at 10 ppm, and the variation of sensor response with change in concentration of CO. (Reprinted with permission from [76]. Copyright 2009, American Chemical Society, Washington, DC, USA).

Another method used to synthesize metal oxides is a low-temperature polymeric citrate precursor (PCP) method. In this method, a multifunctional organic acid (e.g., malic acid, citric acid) chelates with the metal ion and results in the formation of a stable metal complex along with a diol such as ethylene glycol which is called gel. Random distribution of cations starts occurring in the starting solution as a result of gel formation. When this solution is heated to a high temperature, the organic moieties are removed from the gel component, which results in the formation of highly crystalline, very fine, homogeneous oxide powders at lesser temperatures as compared to other solid-state techniques. Jiang et al. [76] employed a simple PCP route for the synthesis of SnO_2_ microstructures followed by a suitable thermal treatment through a surfactant-assisted and solvent-induced assembly technique. In this method, 1.46 g of H_2_C_2_O_4_ was dispersed into a mixed solution containing 40 mL of polyethylene glycol (PEG-600) and 120 mL of ethanol under constant stirring. Further, 1.78 g of SnCl_2_·2H_2_O was added to the solution after H_2_C_2_O_4_ was completely dissolved, followed by dropwise addition of 16 mL of deionized water. After centrifugation and stirring, the obtained product was washed with distilled water and ethanol several times. The products obtained were annealed at 200 °C for 2 h to transform them into flower-like SnO_2_ nano/microstructure morphology. Gas sensing applications of as-obtained SnO_2_ microstructures towards CO and H_2_ reveal excellent sensing properties with an extremely low detecting limit (5 ppm) and notable sensitivity with short response/recovery times and good reproducibility, as shown in Figure 12b,c, which is ascribed to the unique flower-like structure as shown in Figure 12a with three-dimensional geometry of SnO_2_ nanostructures and NPs which is considered as an important constituent for improved gas sensing performance.

**Figure 13:**
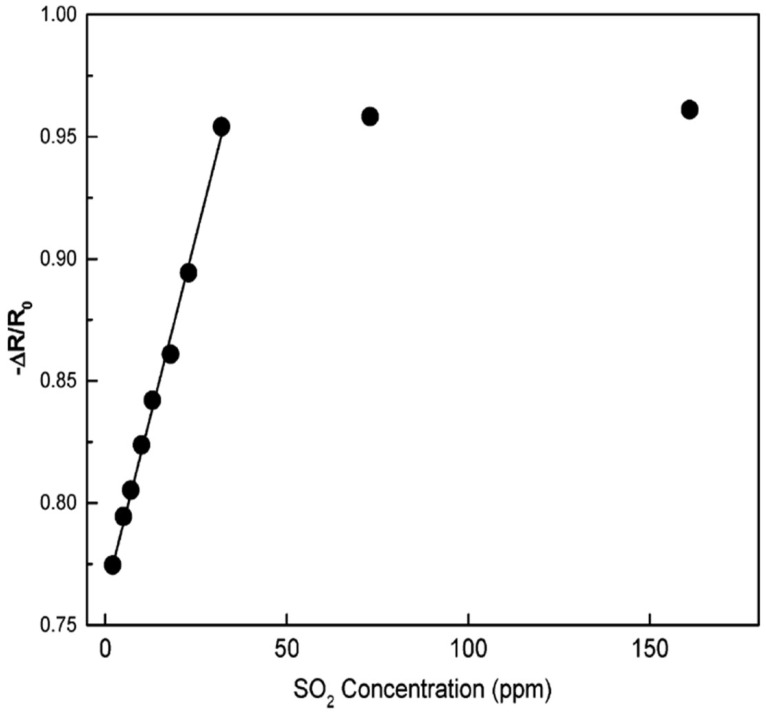
Calibration curve of SnO_2_-1 mol% Ni sensor for relating SO_2_ content to the electrical response. SO_2_ was diluted with dry N_2_ to achieve the desired concentrations. The electrical responses were measured after 4.5 min of injection. (Reprinted with permission from [77]. Copyright 2005, American Chemical Society, Washington, DC, USA).

Hidalgo et al. [77] demonstrated a simple polymeric precursor method for the synthesis of SnO_2_-NiO nanopowders with different compositions. In this method, cationic precursors were added to a citric acid and ethylene glycol solution. Sn_2_-(C_6_O_7_H_4_).H_2_O (tin citrate was prepared by SnCl_2_.H_2_O) and Fe(NO_3_)_3_.9H_2_O were used as the precursors, and HNO_3_ was added to this system to obtain the desired solubilization of citrate ions in the whole system. To obtain the desired molar concentrations, the appropriate amounts of precursors were calculated. After this, the solution was heated at 180–200 °C to promote the polyesterification between ethylene glycol and citric acid resulting in the formation of a polymer chain with sites available to react with the present ions. Then the liquid precursor was heated at 450 °C for 4 h, and a powder rich in carbon was obtained, further ground and then again heat treated at 500 °C for 5 h to guarantee total carbon elimination from the compound with nearly spherical morphology. In the SnO_2_-NiO system, the separation of Ni is used to obtain a rapid sensor response to SO_2_. Compared to pure SnO_2_, response to SO_2_ is enhanced in sensitivity and speed with reliable operation at room temperature for SnO_2_ films containing 1 mol% of Ni. The sensor measurements and drift results showed a completely reversible reaction at this composition with an enhanced electrical response at this temperature. The calibration plots show that the sensor is applicable for detecting SO_2_ with concentrations as low as 25 ppm, as shown in Figure 13.

**Figure 14:**
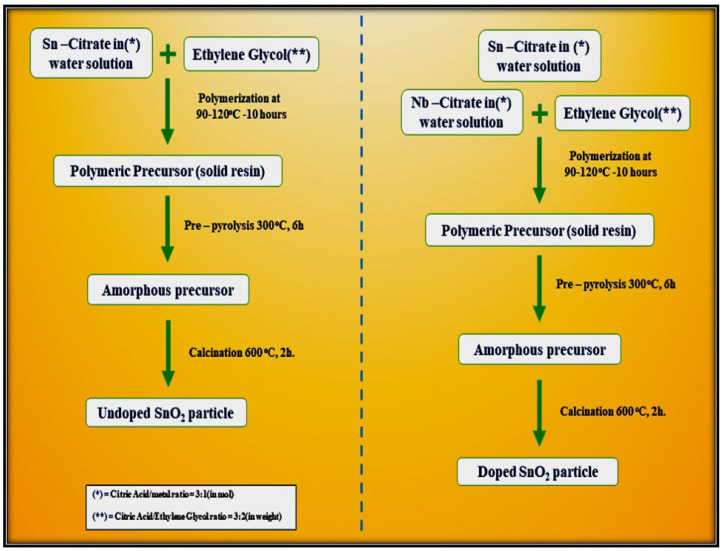
Mechanistic steps involved in the synthesis of undoped SnO_2_ and Nb_2_O_5_–doped SnO_2_ by polymeric precursor method.

Leite et al. [78] demonstrated a simple polymeric route for the synthesis of undoped and Nb_2_O_5_-doped tin oxide. This method is based on the chelation of cations by a hydro carboxylic acid such as citric acid. The citrate solution is then mixed with ethylene glycol through a polyesterification reaction to enhance polymerization. The reaction occurs after the water has been eliminated at a temperature ranging from 90 °C to 120 °C. Figure 14 outlines the steps required for the synthesis of both undoped SnO_2_ particles and SnO_2_ doped with 5 mol.% Nb_2_O_5_. Both the precursors were calcined in two steps, initially at 300 °C for 6 h to promote pre-pyrolysis and then at 600 °C for 2 h to allow complete oxidation of the precursor and to promote crystallization of the SnO_2_ phase, as shown in the flow chart below.

**Figure 15:**
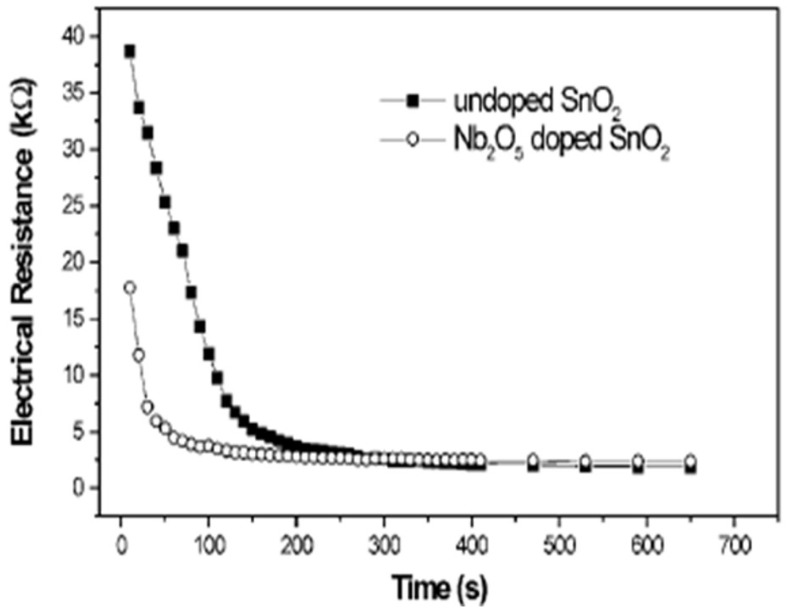
Time response of the Nb_2_O_5_-doped SnO_2_ and undoped particles deposited by spin coating on alumina substrates with ethanol as the testing gas at 100 ppm. (Reprinted with permission from [78]. Copyright 2000, John Wiley and Sons).

Preliminary gas sensing measurements with doped SnO_2_ and undoped SnO_2_ thin films were tested for ethanol. The prepared suspensions were deposited on an alumina substrate by spin coating and were then sintered at 500 °C. Gas sensing tests showed that both powders offer a good response with roughly spherical morphology. However, the sensor response time of doped SnO_2_ was shorter, as shown in Figure 15. The preliminary results depict that doped SnO_2_ has good sensing properties. In other words, Nb_2_O_5_ can be used to control particle size during the synthesis process, which will produce a material with good potential applications in sensor technology.

## 6. Microemulsion or Reverse Micellar Method

**Figure 16:**
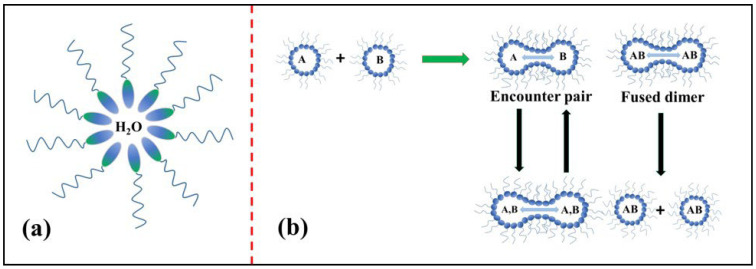
(**a**) Schematic of W/O microemulsion or reverse micelle; (**b**) mechanistic steps involved in the reverse micellar method.

The microemulsion method is another significant procedure for the synthesis of metal oxides; in this method, polar and nonpolar solvents of two immiscible liquids are mixed together by the addition of surfactant in a vessel, which results in the formation of an oil-in-water (O/W) microemulsion [79]. Figure 16a depicts the structure of a microemulsion consisting of a phase encapsulated by a hydrophilic polar head group of a surfactant directed inwards and a chain of long hydrophobic hydrocarbons (which are nonpolar) directed outwards towards the oil phase [80]. In the reverse micelle or microemulsion method, the polar head which is water soluble forms a water pool content that is characterized by a W_0_ ratio, i.e., the concentration of water to the concentration of surfactant. When W_0_ ˂ 15, reverse micelles are formed, and when W_0_ > 15, a microemulsion is formed. The water pool has a size range of 10–15 nm. These reverse micelles have great control over the shape and size of nanoparticles and thus can act as uniformly sized nanoreactors. The important feature of this method is that we can obtain different shapes of micro emulsions in the phase diagram, and the morphologies of the final material, as shown in Figure 16b, can be chosen at different positions in the phase diagram below.

**Figure 17:**
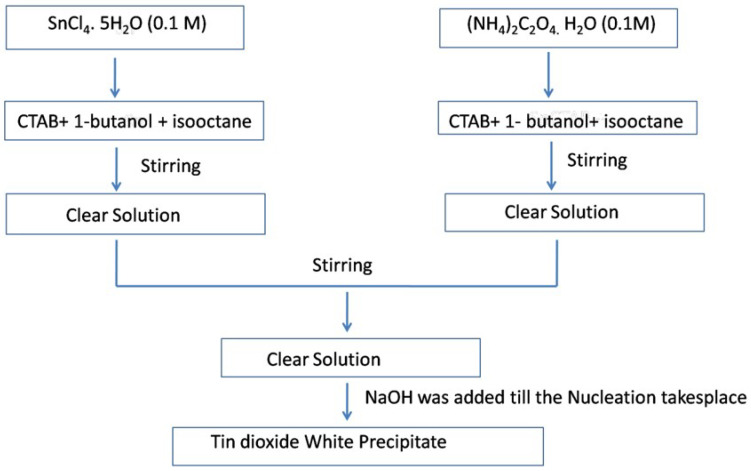
Flow chart for the synthesis of SnO_2_ nanoparticles at 500 °C.

Ahmed et al. [81] used the reverse micellar route to synthesize SnO_2_ nanoparticles, using CTAB as the surfactant. Monophasic tin oxide nanoparticles with rough undefined morphology were found to be crystalline after heating at 500 °C and using NH_3_ as a precipitating agent. Gas sensing measurements of the as-prepared SnO_2_ showed enhanced sensitivity towards n-butanol as compared to the other solution phase techniques such as the co-precipitation method used for the preparation of SnO_2_ polycrystalline samples. The presence of a high oxygen vacancy level in the nanoparticles resulted in enhanced gas sensitivity, as reported in the literature [81]. The schematic procedure for the synthesis of SnO_2_ nanoparticles using reverse micelles is shown in Figure 17.

**Figure 18:**
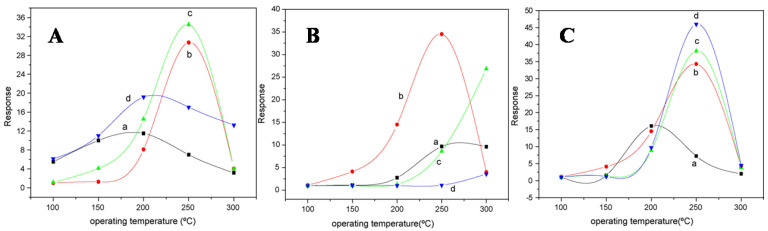
(**A**) Responses of (a) 0, (b) 20, (c) 40 and (d) 60 mol% ZnO nanocomposites calcined at 600 °C to 500 ppm NO_2_; (**B**) responses of 40% ZnO–60% SnO_2_ nanocomposite calcined at (a) 400 °C, (b) 600 °C, (c) 800 °C and (d) 1000 °C to 500 ppm NO_2_; (**C**) responses of 40% ZnO–60% SnO_2_ nanocomposites to (a) 200 ppm, (b) 500 ppm, (c) 800 ppm and (d) 1000 ppm NO_2_. (Reprinted with permission from [83]. Copyright 2008, Elsevier).

Gyger et al. [5] synthesized SnO_2_ nanoparticles by a simple microemulsion method. The preparation of samples was performed via water-in-oil microemulsion (w/o) by mixing 5 mL of hexanol as a co-surfactant and 1.82 gm CTAB as a surfactant in 50 mL of n-dodecane as the nonpolar phase, and further, demineralized water and 2 mL of a 5:1 mixture of methanol were added. In addition, 10 mL of a 0.01 M solution of Sn(Ot-Bu)_4_ (ABCR, 99.99%) in dodecane was added dropwise to this micellar system, and the reaction mixture was left for 12 h to react. This resulted in the hydrolysis at the liquid–liquid phase boundary of the tin alcoholate, due to which polar-phase hollow spheres were established. The reaction was concluded by adding 20 mL of ethylene glycol. The resulting colorless precipitate was washed several times with ethanol followed by centrifugation, and the colorless SnO_2_ nanoparticles were obtained. The nanoparticles obtained with hollow spherical morphology revealed a good sensor response to CO in a concentration range of 50 to 300 ppm that is relevant to the sensing applications. Zhang et al. [82] employed a reverse-micelle-mediated solution route for the synthesis of SnO_2_ one-dimensional nanocrystals. A typical assembly-mediated process was employed for the formation of SnO_2_ hollow spheres. The crystal habit of the SnO_2_ rutile phase and the reverse micelle nature are believed to be responsible for the growth behavior of 1-D SnO_2_ nanocrystals. The temperature range of 220–240 °C resulted in the growth and formation of the morphology of SnO_2_ nanowires, while the morphology of nanorods was obtained when the temperature of the reaction was below 220 °C. The aggregates of SnO_2_ nanocrystals between dendrites and hollow spheres were regarded as the intermediates for the nanowires. The CO gas sensing measurements revealed that the hollow-structured products had a good sensitivity and stability. Compared with SnO_2_ nanorod dendrites and nanowires, the specific surface area is believed to be the dominating factor for enhanced sensing activity. Liangyuan et al. [83] synthesized nanocrystalline ZnO-SnO_2_ nanocomposites as gas sensor materials by successfully employing a microemulsion synthesis route for controlled morphology and grain size. In this method, a water-in-oil microemulsion that contained a maximal amount of water and a minimal amount of surfactant was created. Then CTAB, n-octane, water and n-pentanol were taken in appropriate amounts to form a solution, and the ratio of water or CTAB to alcohol, the precursor salt concentration and the effects of the alcohol chain length on the stability and formation of microemulsions were studied. The morphology of the synthesized nanoparticles was spherical. The performance of the obtained nanocomposites was characterized for gas sensing measurements. The gas sensing results revealed that the obtained nanocomposites are selective for the detection of NO_2_ and CO and are highly sensitive, with sensor response depending on the composite concentration, operating temperature, calcination temperature and concentration of gas in air, as shown in Figure 18a–c. Further, adding surface coatings or dopants of metals or other oxides resulted in a dramatic increase in sensing performance.

## 7. Sol–Gel Method

**Figure 19:**
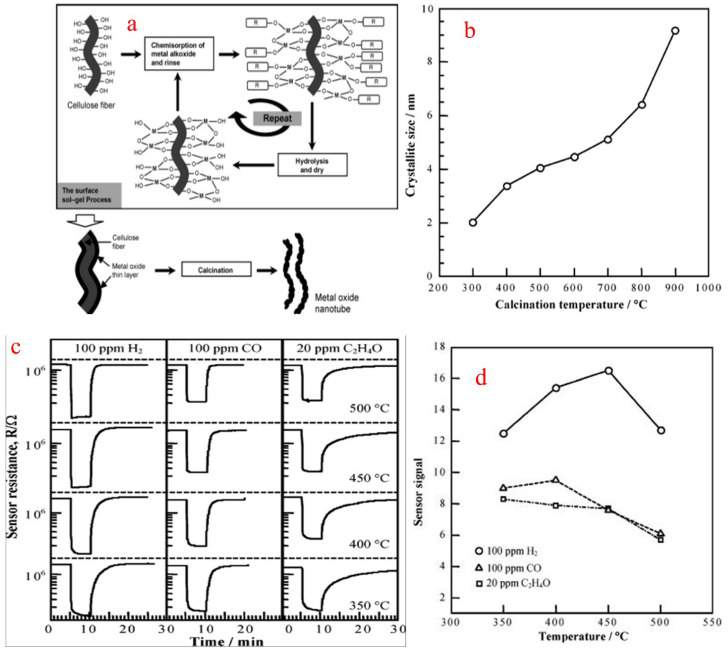
(**a**) Sol–gel synthesis of metal oxide nanotubes using cellulose fibers. (**b**) Dependence of SnO_2_ crystallite size on calcination temperature. (**c**) Response transients of SnO_2_ nanotubes for 100 ppm H_2_, 100 ppm CO and 20 ppm C_2_H_4_O at various temperatures. (**d**) Temperature dependence of SnO_2_ nanotube sensor in response to 100 ppm H_2_, 100 ppm CO and 20 ppm C_2_H_4_O. (Reprinted with permission from [84]. American Chemical Society, Washington, DC, USA).

A sol is a colloidal suspension of a particle in a liquid. In a typical sol–gel process, hydrolysis reactions of a precursor form a colloidal suspension which includes metal–organic compounds such as metal alkoxides or generally inorganic metal salts. The sol–gel process involves the homogeneous solution of one or more selected alkoxides as the starting material because metal alkoxides on hydrolysis give oxide as the colloidal product which remains in the suspension rather than precipitation. In a sol–gel method, the formation of a concentrated suspension, called sol, of a metallic hydroxide is evaporated by dehydration and results in the formation of a semi-solid mass called gel. A varied number of mixed and pure oxides can be obtained by controlled heating of a gelatin material. This method has good control over the particle size and gel. The sol is heated to form gel, and gel on calcination gives the final product. Huang et al. [84] synthesized SnO_2_ nanotubular materials by employing a sol–gel route using a template of a natural cellulosic substance. SnO_2_ gel layers were first coated by a surface sol–gel process using a precursor tetraisopropoxytin-2-propanol adduct to give SnO_2_ with the morphology of nanotubular materials, after calcination in air to form natural hollow replicas of cellulose fibers as shown in Figure 19a. The obtained nanotubes were then calcined at different temperatures, resulting in the formation of different shapes and sizes of nanotubes, as shown in Figure 19b. To obtain pure SnO_2_, a calcination temperature of above 500 °C is needed. The sensor performance for H_2_, CO and ethylene oxide was measured from a sensor setup which was fabricated from the SnO_2_ nanotube sheet, and the sensor signal (S) in response to 100 ppm H_2_ was 16.5 at 450 °C, as shown in Figure 19c,d, and was found to be comparable to that of the SnO_2_ conventional sensor.

**Figure 20:**
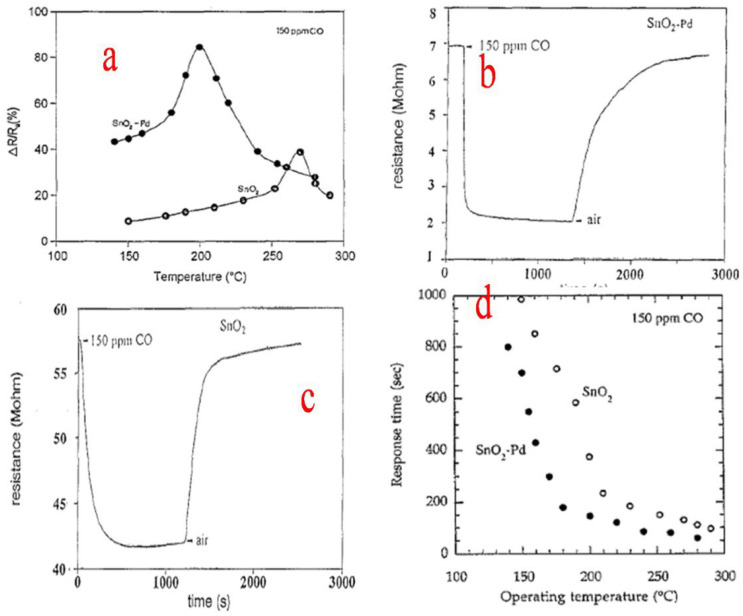
(**a**) Sensitivity as a function of the operating temperature for Pd-modified and pure SnO_2_ thin films. (**b**) Variation in the electrical resistance for a typical Pd-doped SnO_2_ thin film. (**c**) Variation in the electrical resistance for a pure SnO_2_ film. (**d**) Response time versus the operating temperature for both pure and Pd−doped SnO_2_ sensors. (Reprinted with permission from [86]. Copyright 1997, Elsevier).

Zhang et al. [85] used granulated tin to synthesize nanocrystalline SnO_2_ particles by a simple sol–gel method. During the process, the granulated tin in HNO_3_ solution obtained by dissolution is mixed with the citric acid which acts as a stabilizer and slows down the process of condensation and hydrolysis. SnO_2_ nanocrystals obtained ranged from 2.8 to 5.1 nm in size and had a specific surface area ranging from 289 to 143 m^2^g^−1^ when different heating temperatures were employed. The obtained nanocrystallites displayed a reduction in particle size as well as lattice expansion. Further, the precursor condenses and hydrolyzes in an uncontrolled manner in the absence of citric acid, which results in the formation of larger and broader nanocrystals with roughly spherical morphology. This route can be employed to synthesize tin oxide doped nanocrystallites and also serves beneficial purposes in fields such as electronics and engineering and, above all, gas sensing. Rella et al. [86] used a sol–gel technique to synthesize SnO_2_-based thin films. During the process, thin films based on Pd-doped SnO_2_ and undoped SnO_2_ were prepared. Sensing measurements revealed that the sensitivity of the sensor towards CO is increased by the palladium doping, along with a decrease in the sensitivity maximum temperature. The surface area of SnO_2_ is enhanced by Pd, which also helps in catalyzing the oxidation of CO. The changes that occurred in the different aspects of doped and undoped SnO_2_ are shown in Figure 20a–d. Consequently, the increase in the active area to volume ratio and the higher roughness of the modified films led to the better sensing performance of the films loaded with Pd.

## 8. Sensing Applications of Pure and SnO_2_-Based Composites

The change in resistance upon exposure to a particular target gas forms the basis of the underlying working principle mechanism of gas sensors. For an n-type SnO_2_ semiconductor, there is a formation of a depletion layer that is devoid of electrons as oxygen is chemisorbed on the semiconductor surface, which results in the formation of semiconducting resistive and core–shell structures [75,87]; when exposed to the reducing gases, the former is oxidized by the oxygen species carrying negative charge which are adsorbed on the SnO_2_ surface, and thus the sensor shows an increase in conductivity response as a result of the production of more electrons by oxidation reactions. During the process, the first and foremost prerequisite is the development of active materials for gas sensors with ample porosity and large surface areas with ease in the access of analyte gases which increases the sensitivity of sensors. In the case of nanoparticles with dense aggregates, the target gas response is limited by the small pore sizes as compared to the porous nanostructures which have a fast gas response and high surface areas to offer. In general, the packing arrangement of the individual atoms, along with the dimensions of the building blocks and the resultant porosity, plays an important role in the sensing performance of such nanostructures. Moreover, a sufficient surface area is provided by the SnO_2_ hierarchical building blocks for the interfacial chemical reactions to occur as well as the effective diffusion of target gases towards the sensing interface. Further, loading metal onto the surface of prepared porous films results in the enhancement of the selectivity and sensitivity of the gas sensors.

**Figure 21:**
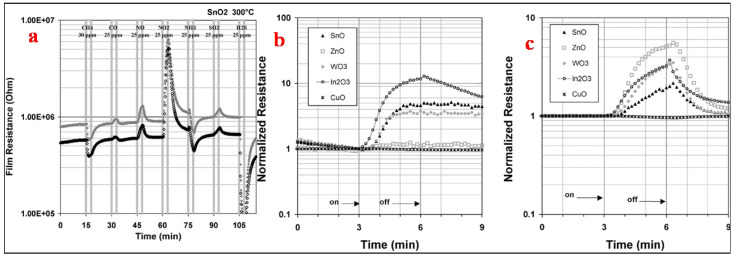
(**a**) Gas exposure sequence used at each temperature to determine the optimal operating temperature and (**b**,**c**) normalized response of SMO sensors to NO_2_ at (a) −200 °C and (b) −400 °C. (Reprinted with permission from [1]. Copyright 2003, Elsevier).

Tomchenko et al. [1] used five thick film semi-conducting metal oxides, namely In_2_O_3_, CuO, SnO_2_, ZnO and WO_3_, to study their gas sensing relative to NH_3_, SO_2_, H_2_S, CH_4_, CO, NO and NO_2_. For thick film sensors, to determine the optimum operating temperatures, the films were studied successively at 200, 300 and 400 °C. The tests of the gas hit sequence used are shown in Figure 21a. In order to maintain steady flow rates in the system, the delivered gas concentration was set at 25 ppm for all gases except for CH_4_, which was kept at 30 ppm. Followed by 12 min of air purge, each gas has a 3 min long exposure. In order to assess the short-term repeatability of the sensor response, this exercise was repeated six times. The obtained maximum sensitivities for this test stage are shown in Figure 21a. All target gases under investigation responded to all types of sensors, thereby showing that the sensors are nonselective in principle. The semiconductors of n-type such as ZnO, WO_3_, In_2_O_3_ and SnO_2_ revealed a decrease in resistance when exposed to CH_4_ and H_2_S and a dramatic increase in resistance when exposed to NO and NO_2_. In contrast, the p-type semiconductors such as CuO showed an increase in resistance for H_2_S and a decrease for NO and NO_2_. The sensitivity even to these active gases was very low for CuO sensors. At 25 ppm of H_2_S, the CuO sensor response was nearly around 1.2 at 300 °C and 400 °C. The magnitudes of other gas responses for CuO were 1.1 (towards NO_2_ at 300 °C) or lower. The target gases were found to be more active towards n-type semiconductors with high sensing of NO_X_ and H_2_S along with the response to other gases of significant interest as well. At 200 °C, a maximum in sensitivity was attained towards particular gases, as can be seen from Table 1. For sensor arrays, 300 °C temperature was chosen as the working temperature because of a compromise between the response speed of the sensor, which became adequate only at 300 °C, and the sharp sensitivity drop observed at temperatures above 300 °C. A comparison of the sensors’ normalized response at 200 °C and 400 °C to NO_2_ is shown in Figure 21b,c which shows that SnO_2_, In_2_O_3_ and WO_3_ sensors demonstrated very slow recovery towards NO_2_ at 200 °C but high sensitivity performance. Remarkable recovery was observed when the same sensors were heated at 400 °C after NO_2_ gas hit them (about 3 min (Figure 21a)), with less sensing sensitivity towards this gas. Taking all these factors and influences into consideration, the operating temperature of 300 °C was selected for sensor materials and other gases of interest with the same tendency for the investigated sensor arrays.

**Figure 22:**
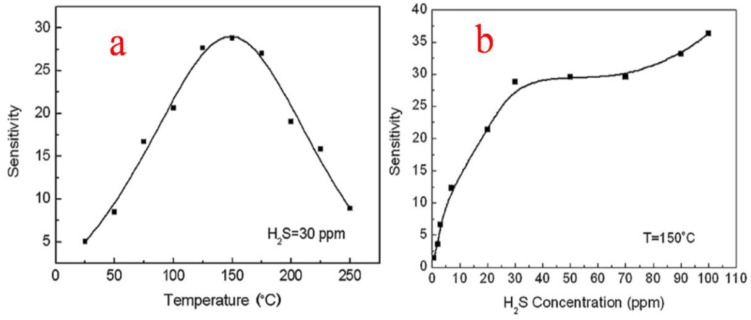
(**a**) Response sensitivity at different operation temperatures for H_2_S at 30 ppm. (**b**) Response sensitivity to different concentrations of H_2_S gas at optimal temperature. (Reprinted with permission from [37]. Copyright 2009, Elsevier).

Liu et al. [37] studied the responses of the thick sensor films at temperatures ranging from room temperature (25 °C) to 250 °C. The sensitivity towards 30 ppm H_2_S was calculated and plotted, and it was observed that at 25 °C for 30 ppm H_2_S, the sensor showed a response with a maximum of around 28.8 at 150 °C, as shown in Figure 22a, and then as the temperature was increased, the sensor response showed a decrease. The enhanced performance of the as-reported SnO_2_ nanoparticles is attributed to the electrical and structural stabilities of the uniform porous micro-structured network of thick film, with more analyte gas penetration and enhanced surface area. This study of thick film sensors thus revealed that the nanoparticles of SnO_2_ with no specific additives were more selective and sensitive in response to even low concentrations (30 ppm) of H_2_S both at room temperature and at optimal detection temperature of 150 °C, as depicted in Figure 22b, showing the high attractiveness and practical applications of SnO_2_ in H_2_S gas sensing.

Tan et al. [38] observed SnO_2_ hollow spheres for gas sensing applications with the aid of templates of carbon microspheres. The as-obtained nanospheres with a size of 100 nm have a significant number of SnO_2_ nanoparticles of nearly 6 nm diameter. The response sensitivity of SnO_2_ hollow-sphere-based sensors to 1000 ppm ethanol is as high as 75, and the recovery and response times are only 10 and 4 s; the sensitivities and recovery times for other ethanol concentrations were lower. Such excellent and enhanced sensing properties can be attributed to the small size of the SnO_2_ hollow spheres and the porous structure of SnO_2_ nanocrystals. On the surface of sensing materials, adsorption and desorption of oxygen cause metal-oxide-based gas sensors to change in resistance, which results in the creation of a depletion layer of electrons on the surface of SnO_2_ nanoparticles. The dependence of the width on the surface depletion layer which operates on the space charge model greatly affects the sensitivity. The surface depletion layer width (L) can be shown in Equations (1) and (2).

L = L_d_(2eV/kT)^½^ (1)

**Figure 23:**
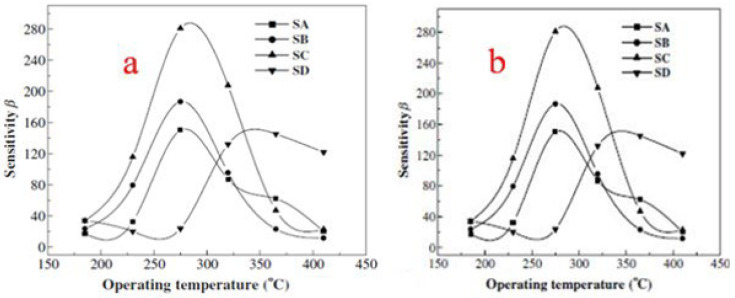
(**a**) The sensitivity of sensors with the effect of different operating temperatures at (**a**) 1000 ppm H_2_ and (**b**) 1000 ppm C_2_H_5_OH. (Reprinted with permission from [45]. Copyright 2003, Elsevier).

L_d_ = (εε_0_kT/2e^2^n_c_)^½^ (2)

where L_d_ is the Debye length, eV is the barrier height, kT is the thermal energy and n_C_ is the carrier concentration. According to the above equation, the calculated L_d_ is about 3 nm for SnO_2_ nanoparticles in this report. The SnO_2_ nanocrystals have a diameter of about 6 nm, which is close to 2L_d_. Now here in the case of the SnO_2_ nanocrystal, due to the adsorption of oxygen in air, the electrons are completely depleted. Consequently, when these sensors are exposed to the reducing gases, the depleted region electrons are released back to the conduction band, due to which the resistance of sensors is sharply changed. This is the reason why theSnO_2_ sensor exhibits high sensitivity. One more reason behind its high sensitivity is that gas molecules diffuse more rapidly in porous structures as compared to denser structures, which balances the desorption and adsorption of target gases quickly and shortens the recovery and response times. The obtained SnO_2_ hollow spheres have a fast response and high sensitivity and are believed to be promising materials for gas sensing applications. Wang et al. [45] prepared mesostructured tin oxide for sensing applications with high specific surface areas of about 368, 343 and 134 m^2^/g for calcination at 300, 350 and 400 °C, respectively. First, in this study, the effect of the operating temperature on the gas sensing properties was studied. As depicted in Figure 23, the operating temperature has a profound effect on the sensors’ sensitivity to 1000 ppm H_2_ (a) and C_2_H_5_OH (b). The sensors obtained from mesostructured tin oxide displayed the highest sensing performance in response to H_2_ at 300 °C. However, the highest sensing performance was observed at 345 °C, as shown in Figure 23a, for the polycrystalline tin oxide based sensor. The sensitivities to H_2_ at 300 °C operating temperature were 13, 13.9 and 23.5 for materials calcined at 300, 350 and 400 °C, respectively. For polycrystalline tin oxide, a maximum sensitivity of 7.3 at 345 °C was achieved, which is lower as compared to the mesostructured tin oxide sensors, which is ascribed to the desorption and adsorption mechanism of gas on SnO_2_ [88,89]. In addition, both the ionic forms of molecular oxygen i.e., O_2_ˉ and Oˉ, are absorbed by the n-type metal oxides. As a result of this fascinating process, the sensor material in presence of the oxygen ion becomes more sensitive to the presence of reducing gases. When the temperature is preferentially low, the surface selectively adsorbs O_2_ˉ, and consequently the sensitivity of the material is small. On contrary, when the temperature is high, the adsorption of Oˉ increases and the sensitivity of the material shows an increase too, as revealed in Figure 23b where the symbols SA, SB, SC and SD represent the sensors based on mesostructured tin oxide calcined at 300, 350 and 400 °C and polycrystalline tin oxide, respectively. On the other hand, there is a steady adsorption of all the oxygen ionic species which were adsorbed earlier when the temperature is increased too much accompanied by a decrease in sensitivity [90]. In addition, the sensing properties are greatly affected by the surface area of SnO_2_ nanoparticles. Consequently, a higher sensitivity to H_2_ was observed for SnO_2_ sensors with higher surface area [91]. In the present investigation, at an operating temperature of 300 °C, the surface area of 136 m^2^/g for mesostructured tin oxide has the highest sensitivity, as shown in Figure 23b. The result obtained was different as compared to the previous studies [92] which suggested that residues of carbon and surfactant on the surface of sensing materials affect the adsorption of reducing gases. The sensing results revealed that mesostructured tin oxide can act as a potential candidate for efficient gas sensing materials.

**Figure 24:**
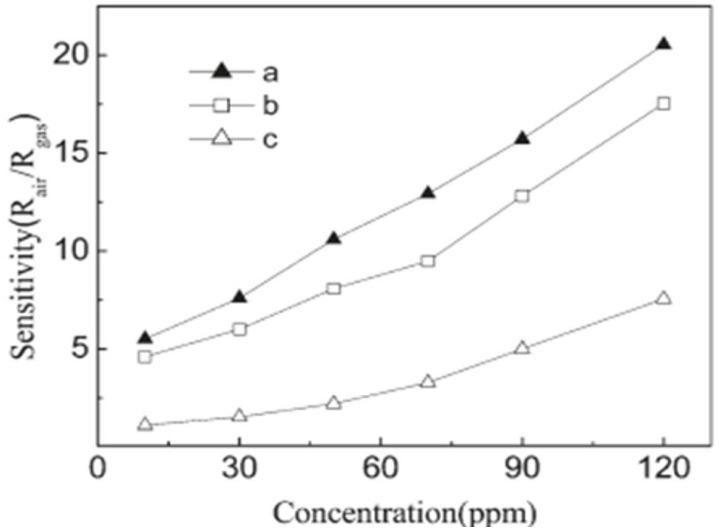
Room temperature sensitivity towards ethanol of (**a**) SnO_2_ nanotubes prepared at 45 °C, (**b**) SnO_2_ nanotubes prepared at 90 °C and (**c**) SnO_2_ nanowires prepared at 45 °C. (Reprinted with permission from [72]. Copyright 2011, American Chemical Society, Washington, DC, USA).

Chiu et al. [70] studied the gas sensing properties of SnO_2_ nanoparticles with an average particle size of about 3.0 ± 0.5 nm. At 25 ppm of ethanol, the sensing measurements of SnO_2_ nanoparticles were carried out to determine the optimum temperature range between 100 and 400 °C. Both the thermally treated and as-synthesized SnO_2_ nanoparticles exhibited different sensing properties due to the different ratios of O/Sn. Further, ethanol was sensed more efficiently by the thermally treated SnO_2_ as compared to the as-reported SnO_2_ in which oxygen sites are occupied by the chloride ions. For the as-synthesized SnO_2_, with the heat treatment of 350 °C for 5 min, the sensing performance can be improved by the exposure of more sites of oxygen and by partly removing Clˉ ions from the nanoparticles. Thus, SnO_2_ nanoparticles treated thermally showed enhanced sensing performance for alcohol at a minimal detection limit of as low as 1.7 ppm. Further, the long carbon chain of alcohol was ascribed as the reason for the enhanced sensor signal. Shi et al. [72] studied the gas sensing property of SnO_2_ nanotubes. It was revealed that the as-prepared SnO_2_ nanotubes with fine grain size and hollow tubular structure may enhance the interaction of the detected gas molecules and the SnO_2_ surface, resulting in full and fast gas access to the nanocrystals of SnO_2_. Hence, the sensor response is expected to show an increase. The selected SnO_2_ nanowires were prepared at 45 °C, and SnO_2_ nanotubes were prepared at 45 °C and 90 °C, and all three samples were then analyzed and evaluated to reveal the gas sensing performance. The gas sensing performance of all three samples showed a gradual increase as the ethanol gas concentration was increased, as revealed in Figure 24. However, the gas sensing measurements revealed that the SnO_2_ nanotubes showed better sensing performance than SnO_2_ nanowires when exposed to ethanol under the same conditions. Further, the improved sensing performance of SnO_2_ nanotubes prepared at 45°C as compared to those prepared at 90 °C was ascribed to the smaller grain size of the SnO_2_ nanotubes obtained at 45 °C. The smaller grain size and hollow tubular surface morphology of SnO_2_ nanotubes as compared to the nanowires were recognized as the reasons for their higher sensing performance. Subsequently, superior gas sensing performance was observed for as-prepared SnO_2_ nanotubes, which proved that these nanotubes can act as efficient and potential candidates for gas sensing applications.

Keeping in view the sensing applications of pristine SnO_2_ nanoparticles, different modifications such as loading of metals on the surface of SnO_2_, doping with different metals and development of different metal oxide/SnO_2_ nanocomposites have been introduced to enhance the sensing activity of SnO_2_ nanoparticles. Suematsu et al. [71] observed that loading Pd onto the preformed SnO_2_ nanocrystals is another significant route for improving the sensing performance of SnO_2_ nanoparticles. Pd-loaded SnO_2_ clustered nanoparticles as sensor devices showed enhanced sensing performance in response to CO and H_2_ when compared with monodispersed nanoparticles. The increase in sensitivity was ascribed to the enhancement in film porosity, as was deduced by the measurements of pore size which revealed that using clustered SnO_2_ nanoparticles results in an increase in the peak pore size of the sensing films, as a result of which the diffusivity of the gas through the sensing films showed an increase. As a consequence, electrical and catalytic sensitization effects produced by the loading of Pd onto the clustered nanoparticles result in an increase in sensor response. More importantly, SnO_2_ nanoparticle clusters loaded with Pd displayed significantly high toluene sensitivity due to the enhanced diffusivity of the analyte gas into the sensing film, at a very low concentration limit of 2.5 ppb.

**Figure 25:**
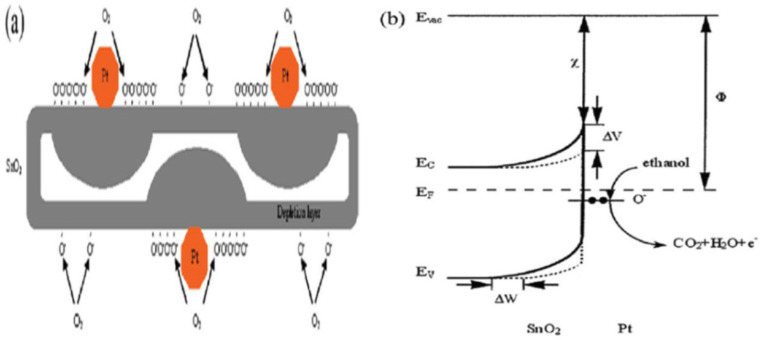
(**a**) Processes taking place at the Pt@SnO_2_ surface, oxygen adsorption on the pristine surface of SnO_2_ nanorods and dissociation and adsorption of oxygen at the regions close to the SnO_2_/Pt interface. (**b**) The energy band diagram of Pt@SnO_2_ nanorods. The solid line depicts the regions close to the SnO_2_/Pt interface, which are wider (∆W) and higher (∆V) than those of the pristine SnO_2_ surface represented by a dashed line. In ethanol atmosphere, the electrons are released easily from the surface reaction at the SnO_2_/Pt interface. (Reprinted with permission from [73]. Copyright 2010, American Chemical Society, Washington, DC, USA).

Xue et al. [73] studied uniformly loaded Pt@SnO_2_ nanorods for gas sensing applications with different concentrations of ethanol, i.e., 10, 50, 100 and 200 ppm, at different working temperatures. The sensitivity measurements of sensors in response to 10, 50, 100 and 200 ppm ethanol reach 3.7, 9.5, 30.1 and 39.5, respectively. Very fast sensor recovery and response times are observed: about 10 and 2 s, respectively. Moreover, good stability and repeatability were observed in the sensors. In addition, the interface between Pt nanoparticles and SnO_2_ nanorods resulted in the improvement of sensing performance [56,93,94,95], which is possibly due to two factors. Firstly, the region close to the interface experiences the possible chemical influence of the catalytic activity of Pt nanoparticles. Secondly, contact between Pt nanoparticles and SnO_2_ nanorods produces an electrical contribution. The chemical nature of Pt influences the chemical effects of the Pt/SnO_2_ interface. Here the role is played by two effects [56,95]. Primarily, oxygen molecules are easily adsorbed on the surface of SnO_2_ nanorods with the assistance of Pt, which is considered a better dissociation catalyst for oxygen as compared to SnO_2_, and thus the oxygen adsorbed on the surface can diffuse faster to create surface vacancies which result in the formation of oxygen ions due to the capture of electrons from the conduction band of SnO_2_ nanorods, as shown in Figure 25a. Moreover, both the molecular-ion conversion rate and the quantity of oxygen adsorbed are increased, which subsequently results in the electron depletion from the SnO_2_ nanorods occurring more rapidly and at a greater degree. As compared to the pristine SnO_2_ surface, the interface depletion layer of SnO_2_/Pt is wider; consequently, at the SnO_2_/Pt interface, the energy band bends wider (∆W), as depicted in Figure 25b. Secondly, hydrocarbons produce more active radicals due to their catalytic property as Pt breaks them down, which ultimately leads to the reaction between reducing ions and surface-adsorbed oxygen ions. Further, at the interface of the SnO_2_/Pt region, the electrons are released more readily from the surface reaction back to the conduction band, which results in the increase in conductivity of Pt@SnO_2_ nanorods in reducing gas atmosphere. Consequently, the sensors are more active in gas sensing in the regions which are close to the SnO_2_/Pt interface. The regions of contact between the Pt interface and SnO_2_ nanorods lead to a greater degree of electron depletion as a case of electrical contribution due to which the work function of SnO_2_ (4.5 eV) is lower than that of Pt (5.65 eV); so, from the SnO_2_ nanorods, the electrons start moving to Pt, which results in the formation of an additional depletion layer and a Schottky barrier at the interface. In addition, at the SnO_2_/Pt interface, the energy band bends higher (∆v). In addition, for explaining the high gas sensing performance of sensors, the small size effect of Pt@SnO_2_ nanorods should be invoked. The depletion layer (L_d_) thickness for n-type metal oxides plays a key role in their sensing performance. When the diameter is close to or smaller than 2L_d_ of metal oxides, it results in high sensitivity as the electrical flow is dominated by the depletion layer. As reported by Xue et al. [73], SnO_2_ nanorods have a diameter that is about 5–10 nm, which is close to 2L_d_ for SnO_2_ materials, for which Ld is about 3 nm. Further, even the relative depletion layer and regions far away from the SnO_2_ interface are also very wide. Thus, all these factors play a significant role in the sensitivity of Pt@SnO_2_ nanorods and consequently result in their high sensing performance. The sensing performance measurements of SnO_2_ nanorods reveal that their sensitivity was enhanced from 21.1 to 39.5 by Pt loading.

Kalmakov et al. [56] studied the performance of gas sensing before and after incorporation of a Pd catalyst on the individual SnO_2_ nanobelts and nanowires configured as gas sensors. It was observed that sensing measurements were carried out in the same reaction chamber in which Pd was deposited in situ, which showed that modification in behavior was due to the functionalization of Pd apart from the change in properties from one nanowire to another. Before metal incorporation, the changes in the conductance revealed that Schottky barrier-type junctions were created on the nanowire surface by the Pd nanoparticles which formed electron depletion regions within the nanowire; thus, the conducting effective channel was constricted, which drastically reduced the conductance. Thus, the dramatic increase in the sensing performance upon the incorporation of Pd was attributed to the combined influence of the “spillover effect” in which atomic oxygen formed catalytically on the Pd nanoparticle surface migrates onto the tin oxide, and the back “spillover effect”, in which the weakly attached molecular oxygen migrates back to the Pd and is dissociated catalytically. Consequently, both precursor capture and delivery of activated ionic species from the surface of the SnO_2_ nanostructure are meticulously enhanced by strategically and catalytically active Pd nanoparticles. Zhang et al. [96] studied Cu-doped and undoped SnO_2_ porous thin films with large surface areas for gas sensing. High selectivity and sensitivity and short response and recovery times were observed for the Cu-doped SnO_2_ porous film gas sensors, which had an operating temperature of 180 °C with average recovery and response times of ~42.4 and ~10.1 in response to 100 ppm of H_2_S, respectively. Further, the sensitivity magnitude of the order of 1 was observed for the Cu-doped SnO_2_ porous film, and it had remarkably better selectivity than the SnO_2_ undoped gas sensor because of the conversion mechanism between CuS and CuO. Further, it was found that H_2_S gas concentration increased linearly from 10 to 100 ppm as the sensor response increased from 2.6 to 25.3. More significantly, these sensors with well-defined porous structures exhibited high reproducibility for sensing due to the precisely controlled process and unique morphology. The results obtained reveal that the fabrication of doped SnO_2_ porous film gas sensors is an efficient way of producing gas sensors with high performance, a lower rate of power consumption and a low cost for H_2_S sensing and can be used generally for the fabrication of gas sensors of semiconductor metal oxides with multilayer, porous and easily doped nanostructures which have strategically important properties for gas sensing.

**Figure 26:**
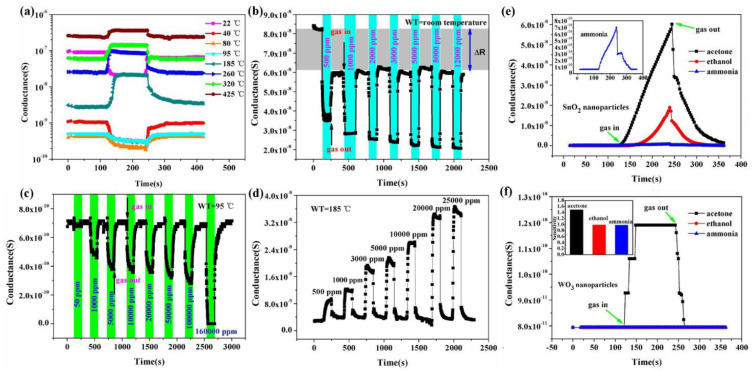
(**a**) Responses of WO_3_−SnO_2_ hollow−sphere−based gas sensors towards 5000 ppm ethanol at various temperatures; (**b**−**d**) the typical sensor response curves to various concentrations of ethanol at room temperature, 95 and 185 °C respectively; (**e**,**f**) the sensing response of SnO_2_ nanoparticles and WO_3_ nanoparticles at 95 °C. (Reprinted with permission from [75]. Copyright 2015, American Chemical Society, Washington, DC, USA).

Another important strategy for improving the gas sensing ability of SnO_2_ is the development of metal oxide nanocomposites. In the literature, several SnO_2_/metal oxide nanocomposites were developed and their application in gas sensing was evaluated. Li et al. [75] studied WO_3_-SnO_2_ hybrid hollow sphere gas sensors with temperature-dependent abnormal p-n transitions. SnO_2_ and WO_3_ are generally well-known classes of materials with n-type semi-conducting nature, whereas the hollow spheres of WO_3_-SnO_2_ with sensing mechanisms controlled by operation temperature display abnormal sensor behavior. In a surprisingly wide operation temperature range, i.e., from room temperature 25 °C to about 95 °C, the sensor showed abnormal p-type responses, while at higher temperatures, it displayed a normal n-type sensing response to ethanol. At 5000 ppm ethanol, the sensor’s conductance response curves at different temperatures were recorded, as shown in Figure 26a. At the temperature of 95 °C, when the sensor is exposed to ethanol with p-type conductance, the sensing response seems to be decreasing rapidly, and when the temperature is kept above 185 °C, an n-type response is shown. Meanwhile, the p-type responses showed a gradual decrease with the increase in the operation temperature. These responses were measured at different ethanol concentrations at temperatures of 22, 85 and 185 °C, as shown in Figure 26b–d. Both n-type and p-type sensor signals were enhanced after ethanol exposure, thereby revealing the possibility that the sensors can be used over a broad range of temperatures. Moreover, at 95 °C, the sensor performance in response to the gases acetone, ammonia and ethanol was observed for WO_3_ and SnO_2_ nanoparticles, as shown in Figure 26e,f. When the sensor was exposed to ammonia and ethanol, normal p-type sensing behavior was displayed, but instead, a higher response to acetone was shown at lower temperatures, thereby showing that SnO_2_ and WO_3_ are more active in response to acetone, resulting in hollow-sphere-based gas sensors of WO_3_-SnO_2_ leading to n-type responses when exposed to acetone. Further, after applying various complex impedance techniques and measuring the sensors’ behavior with various reducing gases, it was revealed that the abnormal sensing response resulted as a consequence of a reaction on the material surface between adsorbed water and the target gas. The competition between extrinsic and intrinsic sensing behavior leads to the control of temperature-controlled n–p switch, which is because protons from the adsorbed water and target gas react with the adsorbed oxygen ions. The total conductivity as an external part is solely regulated by the conduction of the water layer due to the former one, and the intrinsic conductivity of the sensor can be modulated by altering the sensing material’s electron concentration as a result of the latter one. Further, the abundant oxygen vacancies and large area active sites of the hybrid and hollow nanostructures which facilitate the observation of extrinsic sensing behaviors may possibly lead to enhancement of the adsorption of water. This study thus offers new insights into developing humidity-controllable and practical temperature gas sensors with little power consumption based on the extrinsic properties with new approaches concerted towards their sensing mechanisms.

**Figure 27:**
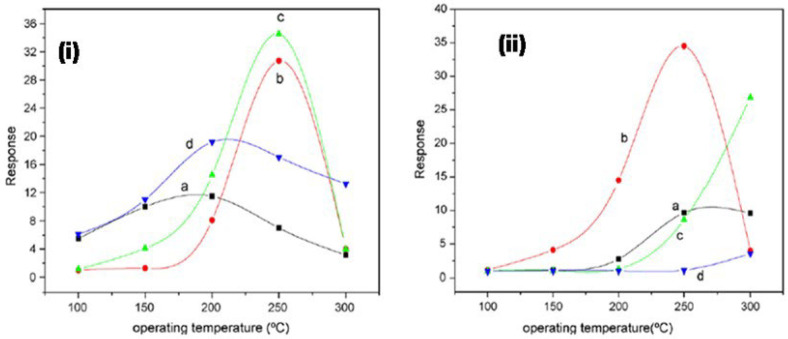
(**i**) The response of different nanocomposites with (a) 0 mol%, (b) 20 mol%, (c) 40 mol% and (d) 60 mol% ZnO calcined at 600 °C to 500 ppm NO_2_; (**ii**) the response of 40% ZnO–60% SnO_2_ nanocomposites calcined at (a) 400 °C, (b) 600 °C, (c) 800 °C and (d) 1000 °C to 500 ppm NO_2_. (Reprinted with permission from [83]. Copyright 2008, Elsevier).

Liangyuan et al. [83] showed that a 40 mol% ZnO–60 mol% SnO_2_ nanocomposite had the maximum sensor response of 34.5 to NO_2_ when calcined at 600 °C with an operating temperature range of 250 °C, while pure nanocrystalline SnO_2_ prepared by the same route had the NO_2_ response of 7.0, as shown in Figure 27i. The enhanced gas response was attributed to the smaller SnO_2_ grain size of 5 nm of the 40% nanocomposite of ZnO as compared to the pure SnO_2_ (19 nm). As the ZnO content in the nanocomposites increased, there was an increase in the NO_2_ sensing response with less than 40 mol% ZnO, but with the higher ZnO loading (˃40 mol% ZnO) there was a decrease in sensing response which revealed that SnO_2_ mainly controlled the NO_2_ response. These results were in concurrence with the studies of surface area measured by BET. The gas response of the nanocomposite showed a decrease with the increase in calcination temperature, which was mainly attributed to the loss ingrain growth size and surface area, as depicted by Figure 27ii. A surface area of around 101 m^2^g^−^^1^ was obtained for the composite with 40% ZnO at 400 °C, which was below the surface area of the sample calcined at a temperature of 600 °C, with lower sensor response, as shown in Figure 27i. Because of this, the nanocomposite does not fit the requirement of the law of crystallinity when heated at 400 °C for sensor applications. On the other hand, there is a decrease in the nanocomposite’s sensor response when it is calcined above 800 °C; this is due to the significant crystal growth of the SnO_2_ nanocomposite, as a result of which there is a decrease in surface area. Thus, it was observed that ZnO-SnO_2_ nanocomposite sensing response is profoundly affected by operating temperature, and in a temperature range of 100 and 300 °C, it was found to be sensitive to NO_2_. At the 250 °C operating temperature, maximum sensing response was observed for NO_2_.

**Figure 28:**
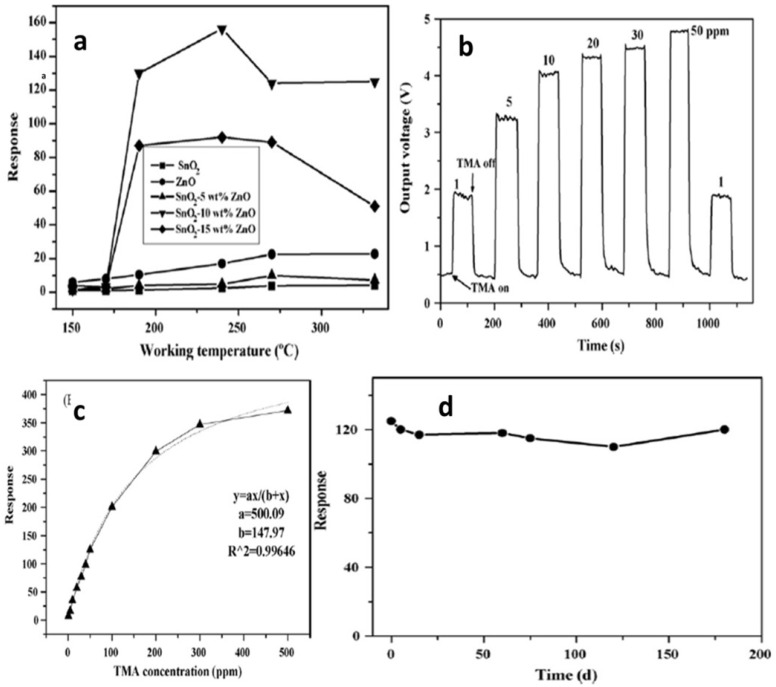
(**a**) The response of sensors to 50 ppm TMA at different working temperatures. (**b**) Response transients and (**c**) magnitude response of 10 wt% ZnO-doped SnO_2_ nanocomposite sensor for different concentrations of TMA at 330 °C. (**d**) The stability of the SnO_2_-ZnO nanocomposite sensor. (Reprinted with permission from [97]. Copyright 2008, Elsevier).

Zhang et al. [97] employed 50 ppm trimethylamine (TMA) at 150–330 °C for measuring sensing response values of SnO_2_-ZnO nanocomposites, SnO_2_ nanoparticles and ZnO microrod sensors, as shown in Figure 28a. At a temperature below 170 °C, the sensing measurement values of 10 and 15 wt% ZnO-doped SnO_2_ were very low, i.e., less than 10, but with the increase in working temperature to 190 °C or above, the sensor response showed a dramatic increase. At 240 °C, a high value of sensor response was obtained for both the types of sensors, i.e., 10 and 15 wt% ZnO-doped SnO_2_ sensors, with sensing values of 156 and 92, respectively. With the increase in temperature for pure SnO_2_ nanoparticles, 5 wt% ZnO-doped SnO_2_ and ZnO microrods, there was a slight increase in sensor response values, which at 330 °C were 4.0, 7.3 and 22.8 respectively. On the other hand, much higher sensor values were obtained at 50 ppm TMA and 190–330 °C for 10 and 15 wt% ZnO-doped SnO_2_ sensors compared to those of pure SnO_2_ nanoparticles, and the response of the 10 wt% ZnO-doped SnO_2_ sensor had the highest value, as shown in Figure 28b–d. By doping with a suitable amount of ZnO microrods, the sensor response to TMA was enhanced greatly for SnO_2_ nanoparticles. In addition, there was an increase in the charge transportation of nanocomposites caused by the addition of one-dimensional ZnO microrods into the SnO_2_ nanoparticles, which resulted in an enhanced sensor response. Further, a quick and high response to TMA at 190–330 °C was observed for the SnO_2_-ZnO nanocomposite sensor. Further, when sensing TMA at 330 °C, the SnO_2_-ZnO sensor displayed some enhanced sensing features such as excellent sensitivity, high selectivity, prompt response/recovery and strong stability. In determining the freshness of a dead fish, this sensor also displayed peculiar and superb sensing characteristics.

**Figure 29:**
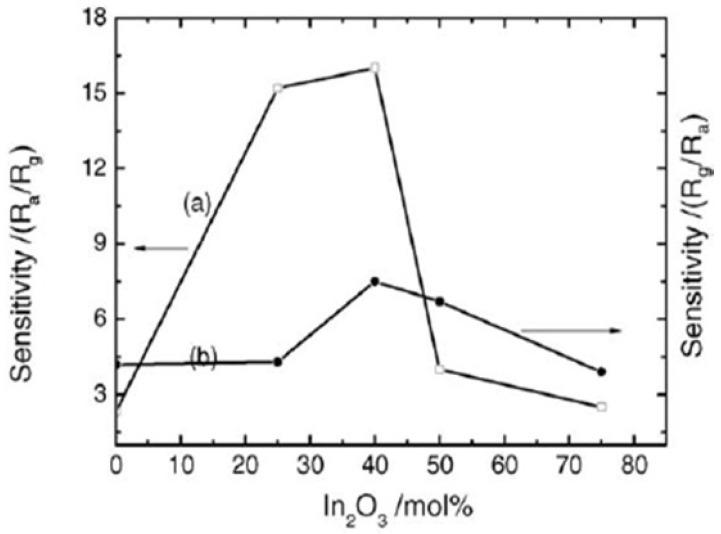
The sensitivity to (**a**) 1000 ppm of CO at 250 °C operating temperature and (**b**) 450 ppm of NO_2_ at 150 °C operating temperature for different nanocomposites calcined at 600 °C. (Reprinted with permission from [98]. Copyright 2006, Elsevier).

Aifan et al. [98] studied the selectivity and sensitivity of SnO_2_-In_2_O_3_ nanocomposites for CO and NO_2_ gases. Further, the effects of the operating temperature and oxide composition on gas sensitivity were studied. Figure 29 depicts the sensor values for NO_2_ at 150 °C and CO at 250 °C with a calcination temperature of 650 °C for different nanocomposites. Nanocomposites containing 40% In_2_O_3_ had the highest sensitivity of 16.0 and 7.5 for CO and NO_2_, respectively, with 0.05 M of total salt concentration and calcination temperature of 600 °C for 4 h. On the other hand, pure nanocrystalline SnO_2_ prepared by the same methodology had sensitivity values of only 2.3 and 4.2 for CO and NO_2_. Moreover, much smaller grain size and high analyte gas adsorption on the surface of nanocomposite resulted in an increase in the sensitivity. Further, selectivity and sensitivity are also affected by the operating temperature of the gas sensor. At a temperature range of 100 to 300 °C, the nanocomposites were found to be sensitive to CO and NO_2_. For CO and NO_2_, 250 °C and 200 °C as optimum operating temperatures reveal the highest sensing performance, respectively. Not only the operating temperature, but also the concentration of gas corresponds to the sensitivity of the 40% In_2_O_3_ nanocomposite. At an operating temperature in air, with the increase in gas concentration, the sensitivity increased linearly. Thus, the above discussion reveals the importance of the application of different gas sensors. It was observed that SnO_2_ shows great potential for application as a sensor. Using different synthetic approaches, the gas sensing property of SnO_2_ can be improved. In addition to the synthesis approaches, different techniques such as development of composites of SnO_2_, doping with different metals and deposition of metals on the surface of SnO_2_ nanoparticles were discussed. It was revealed that these techniques lead to the improvement of the sensing ability of pure SnO_2_ to a considerable extent. The detailed reaction conditions and variation of particle size of SnO_2_ nanoparticles prepared by various methods are represented in Table 2.

## 9. Sensing of CO, CH_4_, NO, NO_2_, NH_3_, SO_2_ and H_2_S by SnO_2_ Nanostructures

The semiconductor metal oxides’ gas sensing ability is very closely associated with the surface of the sensing materials with respect to their capacity of adsorbing oxygen on their surfaces. Tin oxide is an excellent semiconducting material and possesses good electrical properties, as a result of which it can be used for the detection of even low levels of CO gas. The conductance in SnO_2_ is predominantly affected by the number of negatively charged oxygen adsorbates on the surface of the sensing materials as a result of the transfer of electrons to the surface of the SnO_2_ which decreases the electron concentration and increases the resistance of the sensor. At low temperatures, the commonly chemisorbed oxygen ion is O_2_^−^, and at higher temperatures, O^−^ and O^2−^ are commonly chemisorbed [55,149]. When SnO_2_ is brought in contact with an oxidizing gas such as CO, the adsorbed oxygen reacts with the CO gas molecules on the surface of the SnO_2_ material, as a result of which the electrons in the adsorbed oxygen trapped are released again back to the conduction band of SnO_2_, thus resulting in an increase in resistance. The surface of SnO_2_ involves chemical reactions such as the adsorption of CO gas, which is then followed by the desorption of CO_2_ which causes an increase in the conductance; consequently, the surface oxygen vacancies are then replenished due to the adsorption of molecular oxygen. In the case of SnO_2_, the concentration of electrons depends on the stoichiometry deviation determined by the number of oxygen vacancies created by the atomic defects. Further, the electrical properties of SnO_2_ also strongly depend on the surface states which are produced by the oxygen and other gas molecules that are chemisorbed at the grain boundaries due to which space charge appearance and band modulation result in the SnO_2_. Hence, the change in the chemisorbed molecule density is the main factor supposed to be responsible for the electrical response of the SnO_2_ while the tin dioxide phase remains chemically stable. Similarly, other gases such as NO_2_ and NO are adsorbed in the same sensing mechanism in which bridging O vacancies mainly contribute to the sensing. The sensing mechanism in the case of NO_2_ on the SnO_2_ surface is mainly dominated by the adsorption of the single molecule of NO_2_ at the surface of O vacancies. As a result of this, one of the O atoms of NO_2_ fills the vacancy and results in the desorption of the weakly bonded NO from the surface of SnO_2_, as reported by Maiti et al. [150]. Similarly, for the H_2_S gas adsorption on the pure SnO_2_ surface, first, the adsorption of oxygen with the negative charge results in the formation of an electron depletion layer near the surface. Then the oxidation of H_2_S gas at the surface (O^−^_ads_) with the adsorbed oxygen into SO_2_ (g) and H_2_O (g) provides the necessary free electrons to the semiconducting core, thereby increasing the response of the sensor in proportion to the H_2_S concentration. This process requires the diffusion of H_2_S and then its subsequent oxidation. As a result, a very short response time is observed, which indicates that both the gas diffusion and the oxidation reaction are very rapid. Further, the doping of SnO_2_ surface with CuO can show an additional decrease in the sensor response upon exposure to the H_2_S at the interface between CuO and SnO_2_ due to the conversion of semiconducting CuO into metallic CuS [151]. Thus, the short response values were not affected by the doping with CuO. This reveals that the conversion into CuS and the diffusion and subsequent oxidation of H_2_S occur rapidly, which results in the sensing of H_2_S gas. For the sensing of NH_3_ gas, Yuan et al. [152] reported that WO_3_-SnO_2_ nanosheets can significantly sense NH_3_ after coating of SnO_2_ shell layer with WO_3_ through atomic layer deposition. The sensing mechanism is reported to be based on the formation of the depletion layer as well as the built-in electric field at the interface of the WO_3_ and SnO_2_ shell. When the NH_3_ gas comes in contact with the core–shell nanosheet sensors at the surface, the thickness of the electron-depleted region and heterojunction interface of WO_3_@SnO_2_ core–shell nanosheets becomes thinner, thus resulting in decreased resistance and improving the response of the sensor. In brief, the formation of the heterojunction WO_3_@SnO_2_ increases the resistance of the sensor in air, but it further decreases the resistance of the sensor in the NH_3_ atmosphere. As a result of these phenomena, the response of the WO_3_@SnO_2_ hetero structured nanosheet sensor is greatly improved. Hence the formation of the heterojunction as well as the electron-depleted region between the WO_3_ and SnO_2_ nanosheets results in the enhancement of the NH_3_ sensing response. The sensing performance comparison parameters of SnO_2_-based gas sensors using different morphologies are presented in Table 3.

Tin oxide, as a semiconductor metal oxide, can also be employed for the sensing of SO_2_ gas. The origin of the sensing of SO_2_ gas arises from the change in the electrical conductivity of the SnO_2_ sensor as a result of the chemical reactions that take place on the surface of the SnO_2_ due to the physisorbed oxygen species and the target SO_2_ molecules. At the grain boundaries, the atmospheric oxygen molecules are adsorbed and trap the electrons from the interior of the SnO_2_ surface. Hence, at the interface of the SnO_2_, a space charge layer is created with air or between the adjacent grains, which results in the formation of a potential barrier. As a result of the creation of the potential barrier, the easy flow of electrons is impeded by the potential barrier; thereby, the SnO_2_ becomes highly resistive and thus gives a high value of initial resistance. In addition, as the temperature increases, the resistance decreases due to the semiconducting nature of SnO_2_. However, as the temperature is increased to around 160 °C, a small increase in resistance is observed, which is a consequence of the conversion of molecular oxygen (O_2_^−^) into atomic oxygen (2O^−^) and thus the trapping of more free electrons [153,154,155,156]. The interaction with the adsorbed oxygen (O^−^) by the SO_2_ gas molecules on the surface of SnO_2_ releases the trapped charge carriers back. Due to this phenomenon, the resistance of the surface decreases as shown in the following reaction:

SO_2_ + O^−^ (adsorbed) → SO_3_ + e^_^

Thus, in the SnO_2_, the modulation of space charge layer at the grain boundaries and gas molecules (Fermi control energy mechanism) is thus responsible for the change in the sensor resistance to the SO_2_ gas [157,158]. On the surface of SnO_2_, the adsorption of reducing gases such as SO_2_ results in the creation of a new weak donor energy level within the energy band gap [157,159]. Due to this, the resistance on the SnO_2_ surface decreases in the presence of target gas SO_2_. This decrease in the resistance with an increase in the temperature is observed up to a certain temperature and may be due to donor states’ gradual ionization. As the temperature increases, there is an increase in the value of resistance as the rate of desorption of gas molecules is greater than the rate of adsorption. Thus, at higher temperatures, a minimum value of resistance and hence a maximum sensing response is obtained, and this may the possible sensing mechanism for the other types of reducing gases such as NH_3_, H_2_ and H_2_S as well.

The mechanism of CH_4_ sensing was reported by Bonu et al., who concluded from their study that CH_4_ being thermodynamically stable is generally detected at temperatures above 300 °C [160]. From their study, they concluded that the sensing mechanism of reducing gas CH_4_ is mainly attributed to the surface defect density, and the nature of defects and the relative position of defects in the band diagram are considered to be the main factors responsible for the sensing of CH_4_ in the SnO_2_ nanostructures. In addition, without having sufficient surface defects, the high surface-to-volume ratio does not affect the sensing process. Further, the in-plane and bridging “O” vacancy defects in the defect state positions explain the temperature-dependent CH_4_ sensing response. The defect states created close to the conduction band minimum as a result of in-plane “O” vacancies have been found to be very sensitive to the low temperatures due to the very low activation energy as compared to the bridging “O” vacancies. The metal oxides possessing such structures may also exhibit similar effects.

## 10. Conclusions, Challenges and Future Perspectives

In this review, we vividly discussed the synthesis of porous and hollow nanostructures of SnO_2_ and their unique surface morphology and modification by loading and doping with other elements. The progress in the synthetic routes of metal oxides and composites and their status in the field of gas sensing applications have been vehemently discussed. In this report, our main focus was on presenting the research status of tin oxide and its composites and giving an idea and outline to develop them for enhanced gas sensing applications, particularly in environmental remediation. The effects of different reaction conditions on the morphology, structure and other physical aspects of tin oxide and its composites and the effect of various synthetic methods employed were also briefly discussed. The hydrothermal and reverse micellar synthetic routes, with some good and innovative results reported, seem to act as great alternatives to high-temperature conventional synthetic routes. Enhanced gas sensing applications of tin oxide and its composites are reported due to these low-temperature methods which offer better control of reaction conditions to create optimal conditions for their synthesis, giving these sensors a better advantage over their counterparts. These routes are only applicable for the synthesis of cost-effective specific tin oxide and its composites, and thus they are not considered as general routes for synthesis; thus, they serve as an added advantage for these sensors in their synthesis due to their cost-effectiveness and ready availability.

With the advent and advancement of fundamental research, gas sensing serves as the main route to solve the environment-related problems of the world. However, due to increased industrialization and rapid increase in the toxic energy effluents in the environment, tin oxide and its composites still face a number of challenges as gas sensors due to their low sensitivity and selectivity towards which our focus should be concerted. An efficient gas sensor should have the capacity to sense the gases even at very low ppm levels so that even a minimal amount of toxic gases can be detected in the environment. So, in this review, our main focus was on the morphology and particle size of tin oxide and its composites which in turn has a profound effect on the sensitivity, selectivity and stability of the gas sensors. Although many efforts have been concerted, much still needs to be done with regard to fully understanding the mechanism that governs the process of gas sensing. With thorough knowledge for understanding the basic mechanism responsible for gas sensing with the aid of new evolved synthetic strategies and the development of sophisticated characterization techniques, a breakthrough in their gas sensing activity could be expected in the coming future.

In terms of future perspectives, there are still tremendous opportunities to investigate metal oxide nanomaterials and their amazing and boundless combinations with metal oxide nanocomposites for sensor applications. Although the synthetic strategies for tin oxide nanostructures have shown significant development, efforts should still be directed towards better understanding the interactions and underlying sensing mechanisms which are not still clear for tin oxide in particular and metal oxides in general, which in turn affects the sensitivity and selectivity of sensors. Moreover, these hidden sensing phenomena have a role in the design of metal oxides and the optimization of their gas sensing performance. Compared to their pure-phase counterparts, doping meticulously with specific components with desired chemical composition can produce some advantageous characteristics. In addition to the development of tin oxide and its composites as gas sensors, the mass production of these sensors for practical applications should place a special focus on cost-effective techniques for sensor immobilization, techniques with an environmentally friendly approach, improvement in the operating temperature range of gas sensors (sensors should operate at a wide range of temperatures and pH values) and high-yield synthesis of tin oxide and its composites with the exposure of specific properties. For gas sensing applications, other technical problems such as sensitivity, lack of stability and selectivity have resulted in long-term spoiling of the gas response and hence act as the limitations faced by the SnO_2_ sensors and their composites which must be highlighted and addressed as the need of the hour in the sensing field. The presence of various types of structural defects during the synthesis of these materials often led to insufficient quality and low production rates, and hence these materials do not meet the industrial standards for their commercialization. The structural changes introduced during the synthesis can have a large influence on the metal oxides and their composites and consequently on their sensing performance. So, to overcome these limitations of metal oxides and their composites in the gas sensing field, using synthetic chemical methods, desired morphological features can be tuned so that the surface of the material can be easily accessible to the particular target gas, which can in turn have a profound effect on the sensing parameters such as selectivity, sensitivity and stability, and the quality assurance and quality control of the sensors can also be enhanced through these proactive and selective morphological changes being incorporated into the metal oxides and their composites, which can lead to the substantial enhancements in these parameters in the future developments of sensors. The future pathway and strategies should further include the miniaturization of structural and chemical approaches with the incorporation of desired structural changes in a highly precise manner, which is still an unresolved challenge that requires further research, innovation and development. Above all, the present review will further elevate the hope to stimulate the progress and development of tin oxide and its composites with concerted efforts and strategies to be employed by researchers to achieve excellent sensing performance with good stability, high sensitivity and selectivity and environmental friendliness.

In summary, we have reviewed the recent advances in the preparatory methods, in particular, the hydrothermal and reverse micellar synthetic routes, for the tin oxide hybrid materials with enhanced and optimized control over the structural features which further led to the advancement of this unique group of materials. The engineering of unique structural features such as morphology, size, engineered defects, functionalization, crystallinity and doping which consequently leads to the precise miniaturization of the various physicochemical properties has produced miraculous results in using tin oxide and its composites in the field of gas sensing to develop particular specific gas sensors, as has been discussed above. In addition, the structural and compositional features have been effectively probed as a result of the improvements in the integration and characterization methods which have helped in better understanding the structure–property relationships within these materials. Furthermore, we discussed the sensing mechanisms and the processes governing them for the development of sensing platforms, highlighting the basic and fundamental principles governing their response, sensitivity and selectivity. In addition, emphasis has also been placed on how the compositional and structural features and the surface chemistry of the tin oxide and its hybrids play a crucial role in their electrical properties which ultimately are revealed in the form of fascinating applications such as the one in the field of gas sensing. Furthermore, tin oxide and its composites have found very wide applications in the gas sensing area due to their various physical and chemical properties such as large surface-to-volume ratio, tunable band gaps and excellent thermal stability which has been widely harnessed in the sensing field. Last but not least, the advancements in the recent past in the preparatory methods for tin oxide and its composites have been discussed, showing how an even greater degree of control over their structural and morphological features such as crystallinity, size, thickness, functionalization and doping has been achieved. Noteworthily, tin oxide and its hybrids with specific advances in the field of gas sensing were vividly probed and illustrated in the context of three major analyte are as, namely gases, ions and volatile organic compounds, with a detailed discussion of both historical and analytical perspectives.

## Figures and Tables

**Table 1:** Maximum sensitivities during the experiments on sensor operating temperatures. Here a negative value means that the sensor’s resistance decreased during the gas hit. If the resistance increased, the corresponding sensitivity is shown as a positive number.

Sensor	Temperature (°C)	Sensitivity (S)						
		CH_4_	CO	NO	NO_2_	NH_3_	SO_2_	H_2_S
SnO_2_	200	−1.18 *	1.40	9.39	4.59	−1.72	1.23	−38.85
	300	−1.30	1.08	2.48	6.13	−1.33	1.13	−14.73
	400	−1.27	−1.09	1.09	1.95	−1.20	−1.03	−6.69
ZnO	200	1.04	3.00	3.53	1.11	−1.10	1.08	−16.90
	300	−1.25	1.56	9.40	11.00	1.20	1.50	−21.67
	400	−1.24	1.01	1.59	5.12	1.08	1.04	−13.16
WO_3_	200	−1.10	1.20	8.92	3.73	−1.42	1.3	−34.11
	300	−1.16	1.03	2.56	4.53	−1.04	1.07	−28.18
	400	−1.14	1.01	1.18	3.11	1.11	1.02	−14.02
In_2_O_3_	200	−1.04	1.56	17.00	6.66	−1.99	1.53	−43.08
	300	−1.04	1.06	1.85	3.42	1.22	1.03	−6.98
	400	−1.02	1.02	1.09	1.40	1.08	−1.01	−2.60
CuO	200	1.01	1.01	−1.03	−1.03	1.02	−1.01	1.16
	300	1.03	1.01	−1.03	−1.09	1.04	1.01	1.18
	400	1.02	1.01	−1.01	−1.04	1.07	1.01	1.24

* indicates that the sensors response decreased during the gas hit.

**Table 2:** Reaction conditions and variation of particle size of SnO_2_ nanoparticles prepared by various methods.

Material	Solvent	Precursor	Conditions	Description	Ref.
Hydrothermal Method					
SnO_2_nanosheets	Ethanol and water	SnCl_2_.2H_2_O	120 °C for 6 h	Rutile structure with size <100 nm	[99]
SnO_2_@ carbon hollow nanospheres	Ethanol and water	Urea, silica nanospheres and K_2_SnO_3_.3H_2_O	150–190 °C for 36 h	Tetragonal rutile SnO_2_ with 30 nm thickness	[100]
SnO_2_ nanoparticles and nanorods	Water	SnCl_2_.2H_2_O	130–160 °C, calcination at 350 °C	Rutile structure with 4–6 nm size	[101]
SnO_2_/α-Fe_2_O_3_ semiconductor nanoheterostructures	Water	Fe_2_O_3_, SnCl_4_.5H_2_O, NaOH	Heated at 220 °C and drying at 50 °C for 4 h	Tetragonal SnO_2_ phase with 5 nm size	[102]
SnO_2_ nanosheets	Ethanol and water	SnCl_2_.2H_2_O and NaOH	180 °C/12 h, vacuum dried at 80 °C/1 h	Rutile SnO_2_ NPs with 5 nm diameter	[103]
SnO_2_	Water	SnCl_4_.5H_2_O and sucrose	600 °C for 3 h	Rutile structure with 10 nm size	[89]
SnO_2_	Ethanol and water	SnCl_4_.5H_2_O and NaOH	170–190 °C	Rutile structure (size 70–105 nm)	[104]
SnO_2_	Water	CTAB, NaOH and SnCl_4_.2H_2_O	400 °C for 2 h	Rutile SnO_2_ with 42 nm size	[105]
SnO_2_ nanowires	Water	SnCl_4_.5H_2_O and NH_4_(OH)	370 °C for 5 min	Rutile, diameter 70–150 nm, length 20–100 µm	[106]
SnO_2_ nanorods	Water	SnCl_4_, NaOH and CTAB	Heated at 160 °C for 12 h	Rutile, diameter 40–100 nm, and 2–3 µm in length	[43]
SnO_2_ microspheres	Water	SnO_2_ methenamine, carbamide and sodium hydrate	Heated at 160 °C for 16 h	Tetragonal structure, 0.5–1 µm diameter	[107]
SnO_2_ nanorods	Water	SnCl_4_, HCl and NH_4_(OH)	Heated at 95 °C for 15 min	Tetragonal with diameter of 100–150 nm and length of 1–2 µm	[108]
SnO_2_ nanocolloids	Glucose	K_2_SnO_3_.3H_2_O, glucose	350–500 °C for 1 h	Tetragonal, mean size 9 nm	[109]
SnO_2_ quantum dots	Water	SnCl_4_.5H_2_O and hydrazine hydrate	Heated at 150 °C for 24 h	Tetragonal, 3 nm particle size	[110]
SnO_2_ nanorods	Mixture of heptane and hexanol	Sodium dodecyl sulfate, SnCl_4_ and NaOH	Heated at 200 °C for 18 h	Rutile, diameter 8–15 nm, length 150–200 nm	[111]
SnO_2_–V_2_O_5_ CNT	Water	NH_4_VO_3_, SnCl_2_.2H_2_O and urea	Heated at 500 °C for 3 h	Crystalline structure with 10 nm particle size	[112]
Zn-doped SnO_2_ nanoflowers	Ethanol and water	Zn (CH_3_COO)_2_. 2H_2_O, SnCl_4_ and NaOH	Heated at 180 °C for 24 h	Tetragonal SnO_2_ with uniform size of 1 µm	[113]
SnS_2_/SnO_2_ non-heterojunction	Water	SnCl_2_.5H_2_O, CH_3_CSNH_2_	190 °C for 6 h	Tetragonal SnO_2_ NPs with sizes 3–6 nm and hexagonal SnS_2_ nanoflakes with sizes 22–55 nm	[114]
Ni/SnO_2_ core-shell	Water and ammonia	1,2-Propanediol, SnCl_2._2H_2_O, H_2_O_2_	Heated at 120 °C for 15 h	Rutile structure with 0.7–0.95 µm diameter	[115]
CuO/SnO_2_ core-shell	Ethanol and water	SnCl_4_, NaOH, Cu (NO_3_)_2_.2H_2_O	Heated at 190 °C for 24 h and then heated at 800 °C for 2 h	Tenorite SnO_2_ nanorods of diameter 10 nm and length 100 nm and size of CuO NPs as 4 nm	[116]
SnO_2_ nanowires	Ethanol	SnCl_4._5H_2_O, NaOH	Heated at 285 °C for 24 h	Rutile, diameter 80 ± 5 nm and length of ~2.5 ± 0.1 µm	[117]
SnO_2_ nanotubes	Ethanol and water	Na_2_SnO_3_.5H_2_O, urea	600 °C under Ar for 1 h	Nanotubes (100–300 nm)	[118]
SnO_2_	Water	H_2_SO_4_, H_2_O_2_ and tin powder	Heated at 150 °C for 6 h	Tetragonal SnO_2_ with size 2.9 nm	[119]
SnO_2_ nanocrystals	Water and ethanol	SnCl_2._2H_2_O, SDS, PVP, NaOH, TPAB, CTAB	Heated at 180 °C for 12 h	Tetragonal rutile SnO_2_ with 10–20 nm grain size	[120]
SnO_2_ nanostructures	Water	SnCl_4_, triethylenediamine	Heated at 200 °C for 40 h	Tetragonal with size distributions 3–8 nm	[121]
SnO_2_ hollow nanospheres	Water	D-Glucose monohydrate, SnCl_2_, glucose	Heated at 500 °C for 5 h	Tetragonal, sizes of 15 and 60 nm at different concentrations	[122]
Mn-doped SnO_2_ DMS nanoparticles	Water	SnCl_2_. 2H_2_O, (CH_3_COO)_2_Mn.4H_2_O, (NH_4_)_2_C_2_O_4_.H_2_O	Refluxed at 70 °C for 12 h and vacuum dried at 55 °C for 1 h	Tetragonal SnO_2_ with particle size distribution in the range of 5–11 nm.	[123]
Co-doped SnO_2_ DMS nanoparticles	Water and ethanol	SnCl_2_.2H_2_O, cobalt acetate tetrahydrate, diammonium, oxalate	Refluxed for 12 h at 70 °C and vacuum dried at 60 °C	Tetragonal SnO_2_ with average size of 8–13 nm.	[124]
Ni-doped SnO_2_ DMS nanoparticles	Water and ethanol	SnCl_2_.2H_2_O, nickel chloride hexahydrate, (NH_4_)_2_C_2_O_4_	Refluxed at 70 °C for 12 h and dried at 60 °C	Tetragonal with particle size in the range of 8–12 nm	[125]
Polymeric Method					
SnO_2_	Water	SnCl_2_.2H_2_O, citric acid	Heated at 300 °C for 6 h	Tetragonal with size of 113.8 nm	[126]
SnO_2_/Sb_2_O_3_	Water	SnCl_2_, citric acid, ethylene glycol, HNO_3_, Sb_2_O_3_	Heated at 300 °C for 2 h	Cassiterite structure with different crystalline size	[127]
SnO_2_ and Sb-doped SnO_2_	Ethylene glycol	SnCl_2_.2H_2_O, SnCl_4_.5H_2_O, Sb_2_O_3_, HNO_3_, citric acid	Heated at 90 °C for 4 h	Cassiterite type tetragonal structure 20 nm in size	[128]
Sb-doped SnO_2_ thin films	Ethylene glycol	Citric acid, tin and antimony tartarate	Heated at 550 °C for 1 h	Cassiterite structure with varying sizes from 3.5 to 9 nm	[129]
SnO_2_	Water and ethylene glycol	SnCl_2_.2H_2_O, citric acid	Temperatures from 500 to 900 °C for 2 h	Tetragonal with particle size of ~20 nm	[130]
SnO_2_	Ethylene glycol	SnCl_2_.2H_2_O, citric acid	Heated at 400 °C/12 h	Tetragonal, size range 24–86 nm	[131]
NiO/SnO_2_ and Fe_2_O_3_/SnO_2_	Ethylene glycol	SnCl_2_.2H_2_O, citric acid, Fe(NO_3_)_3_.9H_2_O, Ni(NO_3_)_2_.6H_2_O	Heated at 500 °C for 15 h	Tetragonal phase	[132]
Pure and Ce-doped SnO_2_	Ethylene glycol	SnCl_2_.2H_2_O, citric acid, HNO_3_, Ce(NO_3_).6H_2_O	Heated at 400 °C for 4 h	Tetragonal rutile structure with particle size of 20 nm	[133]
Pure and Ni-doped SnO_2_	Ethylene glycol	SnCl_2_.2H_2_O, Ni(NO_3_)_2_, HNO_3_, citric acid	Heated at 500 °C for 15 h	Rutile type phase of SnO_2_ with 13 nm particle size	[134]
SnO_2_	Ethylene glycol	SnCl_2_.2H_2_O, HNO_3_, MgO Fe(NO_3_)_3_.9H_2_O	Heated at 900 °C for 15 h	Tetragonal nanostructures with smaller particle size	[135]
Reverse Micellar Method					
ZnO-SnO_2_ nanospheres	CTAB, n-pentanol, n-octane	Zn^2+^ and Sn^4+^salts as precursors	Calcined at 400–1000 °C for 6 h	Tetragonal with size ranging from 5 to 15 nm	[83]
PdO-SnO_2_ nanoparticles	Cyclo hexane,	Sn(CH_3_COO)4, Pd (NO_3_)_2_	Calcined at 600 °C for 3 h	Tetragonal structure with particle size of 10 nm	[136]
SnO_2_ nanoparticles	Cyclo hexane, n-butanol, isopropanol	Tin isopropoxide	Calcined at 500 °C for 2 h	Tetragonal structure with particle size 2–10 m	[137]
SnO_2_ nanoparticles	1-Butanol, iso-octane	SnCl_4_.5H_2_O	Calcined at 500 °C for 5 h	Crystalline structure with particle sizes of 70 and 150 nm	[81]
SnO_2_ nanoparticles	Hexane	SnCl_4_.5H_2_O	Calcined at 650 °C for 5 h	Tetragonal structure with average particle size of 10–16 nm	[138]
SnO_2_ nanoparticles	Hexanol, heptane	Sn(OH)_6_^2−^	Heated at 60–70 °C for 5 h	Tetragonal rutile structure with particle size of 5–10 nm	[82]
SnO_2_ nanoparticles	Ethanol	SnCl_2_, KClO_3_	Heated at room temperature overnight	Tetragonal structure with particle size 80–120 nm	[6]
SnO_2_ nanocrystals	Heptane, hexanol	Sn(OH)_6_^2−^	Heated at 60–70 °C for 5 h	Tetragonal with rutile structure with particle size in the range of 6–22 nm	[82]
Sol–Gel Method					
SnO_2_ nanostructures	Citric acid and polyethylene glycol	Zn(NO_3_)_2_.6H_2_O	600 °C for 4 h	Hexagonal wurtzite with mean sizes of 35 nm	[139]
SnO_2_/AgO_2_ nanoparticles	Ammonia solution and water	SnCl_2_.5H_2_O and AgNO_3_	Annealed at 100, 300 and 500 °C	Crystalline with mean particle size of 23, 48 and 78 nm	[140]
SnO_2_/TiO_2_ nanoparticles	Water and isopropyl alcohol	Sncl.5H_2_O titanium isopropoxide	Calcined at 540 °C for 2 h	Tetragonal structure with particle size of ∼8 nm	[141]
Au SnO_2_ nanoparticles	Citric acid and water	SnCl_4_.5H_2_O, HAuCl_4_.4H_2_O	Heated at 500 °C for 2 h	Crystalline structure with particle sizes of 50 and 30 nm	[142]
MWCNT/SnO_2_ nanoparticles	Water	SnCl_4_.5H_2_O, MnCl_4_.4H_2_O, H_2_C_2_O_4_, MnCl_4_.5H_2_O, NH_3_.H_2_O, citric acid	Calcined at 550 °C for 3 h	Crystalline structure with particle size ranging from 10 to 20 nm	[143]
SnO_2_ /Co_3_O_4_ nanospheres	Methanol	SnCl_2_.2H_2_O, C_2_H_7_NO, C_4_H_6_CoO_4_⋅4H_2_O	Room temperature for 24 h	Tetragonal rutile structure	[144]
Cd doped TiO_2_/SnO_2_ nanoparticles	Ammonium hydroxide, water	SnCl_4_.5H_2_O, tetra butyl Ti, CdNO_2_	Sintered at 773 K for 3 h	Crystalline with sizes in the range of 30–32 nm.	[145]
ZnO/SnO_2_ nanostructures	Ethanol	Zinc acetate dehydrate, tin (II) 2-ethyl hexanoate, triethanolamine	Heated at 350 °C for 1 h	Amorphous structure with 31 nm grain size	[146]
SnO_2_/TiO_2_ microstructures	Isopropyl alcohol, tetra methyl ammonium hydroxide, ethanol	Tetra-methyl ammonium hydroxide, SnCl_4_.5H_2_O, NH_3_ solution, AgNO_3_	Calcined at 450 °C for 2 h	Anatase phase with particle size of 30 nm	[147]
Polyaniline/SnO_2_ nanoparticles	Ethanol, water	SnCl_2_.2H_2_O, ammonium peroxydisulfate, aniline	Calcined at 400 °C for 2 h	Tetragonal with particle size in the range of 5–9 nm	[148]

**Table 3:** Comparison of sensing performance parameters of SnO_2_-based gas sensors with different morphologies.

Composition	Material Morphology	Operating Temperature	Target Gas/Concentration (ppm)	Response	Ref
SnO_2_	Nanoparticles	200 °C 300 °C	CO/25 ppm NO_2_/25 ppm	1.40 6.13	[1]
SnO_2_	Spherical NPs	150 °C	H_2_S/30 ppm	28.8	[10]
SnO_2_	Hollow spheres	350–300 °C	CO/20–290 ppm	Good response	[5]
SnO_2_	Hollow spheres	Room temperature	Ethanol	6.8	[6]
SnO_2_	Nanosheets	150 °C	H_2_S/30 ppm	28.8	[37]
SnO_2_	Hollow spheres	300 °C	Ethanol/1000 ppm	75	[38]
SnO_2_	Nanosheets	275 °C	Ethanol/100 ppm	56.2	[39]
Pd/SnO_2_	Clustered nanoparticles	300 °C	CO/200 ppm H_2_/200 ppm Toluene/50 ppm	1350 2020 1720	[71]
Pt/SnO_2_	Nanorods	300 °C	Ethanol/200 ppm	39.5	[73]
Sc/SnO_2_	Nanoparticles	300–400 °C	CO/1000 ppm	16	[74]
WO_3_/SnO_2_	Hollow nanospheres	P-type; room temperature to 95 °C N-type; above 185 °C	Ethanol/5000 ppm Ammonia/500 ppm CO, H_2_ and NO showed no response	Normal response	[75]
CuO/SnO_2_	Nanorods	60 °C	H_2_S/10 ppm	9.4 × 10^6^	[116]
Pt@SnO_2_	Nanoparticles	160 °C	CO/400 ppm	450	[94]
SnO_2_ doped with Pt and Pd	Nanoparticles	450 °C	CO/500 ppm	8.5	[95]

## Data Availability

Not applicable.
